# Phosphopeptide binding to the N-SH2 domain of tyrosine phosphatase SHP2 correlates with the unzipping of its central β-sheet

**DOI:** 10.1016/j.csbj.2024.02.023

**Published:** 2024-03-02

**Authors:** Michelangelo Marasco, John Kirkpatrick, Teresa Carlomagno, Jochen S. Hub, Massimiliano Anselmi

**Affiliations:** a Molecular Pharmacology Program, Memorial Sloan Kettering Cancer Center, New York, NY, USA; b School of Biosciences, University of Birmingham, Edgbaston, B15 2TT Birmingham, UK; c Institute of Cancer and Genomic Sciences, University of Birmingham, Edgbaston, B15 2TT Birmingham, UK; d Theoretical Physics and Center for Biophysics, Saarland University, 66123 Saarbrücken, Germany

**Keywords:** SHP2 phosphatase, N-SH2 domain, Molecular dynamics simulations, NMR spectroscopy, Allosteric coupling, Protein flexibility

## Abstract

SHP2 is a tyrosine phosphatase that plays a regulatory role in multiple intracellular signaling cascades and is known to be oncogenic in certain contexts. In the absence of effectors, SHP2 adopts an autoinhibited conformation with its N-SH2 domain blocking the active site. Given the key role of N-SH2 in regulating SHP2, this domain has been extensively studied, often by X-ray crystallography. Using a combination of structural analyses and molecular dynamics (MD) simulations we show that the crystallographic environment can significantly influence the structure of the isolated N-SH2 domain, resulting in misleading interpretations. As an orthogonal method to X-ray crystallography, we use a combination of NMR spectroscopy and MD simulations to accurately determine the conformation of apo N-SH2 in solution. In contrast to earlier reports based on crystallographic data, our results indicate that apo N-SH2 in solution primarily adopts a conformation with a fully zipped central β-sheet, and that partial unzipping of this β-sheet is promoted by binding of either phosphopeptides or even phosphate/sulfate ions.

## Graphical Abstract

**Figure:**
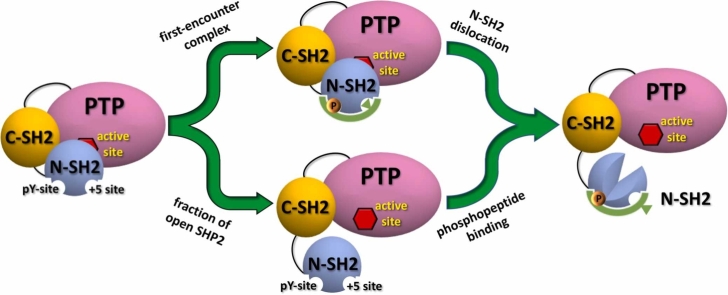

## 1 Introduction

Src homology-2-containing protein tyrosine phosphatase 2 (SHP2) is a ubiquitously expressed, non-receptor protein tyrosine phosphatase encoded by the *PTPN11* gene [1], [2], which plays an important role in cell proliferation, differentiation, migration, and apoptosis, and is involved in multiple intracellular signaling cascades, such as the RAS/MAPK [3], [4], [5], PI3K/AKT [6], [7], Jak/STAT [8], [9], and PD-1/PD-L1 pathways [10], [11]. Germline mutations of *PTPN11* have been identified as the most common cause of two inherited disorders [12], namely Noonan syndrome [13], [14] and Noonan syndrome with multiple lentigines (formerly known as LEOPARD syndrome) [15], [16]. Somatic gain-of-function mutations of *PTPN11* have been linked to several hematological malignancies, such as juvenile myelomonocytic leukemia (JMML) [17], [18], and to several types of human solid malignancies [19], [20], [21]. Currently, SHP2 represents a key target for anticancer therapy [22], [23], [24], [25], [26], [27], [28], [29].

**Fig. 1:**
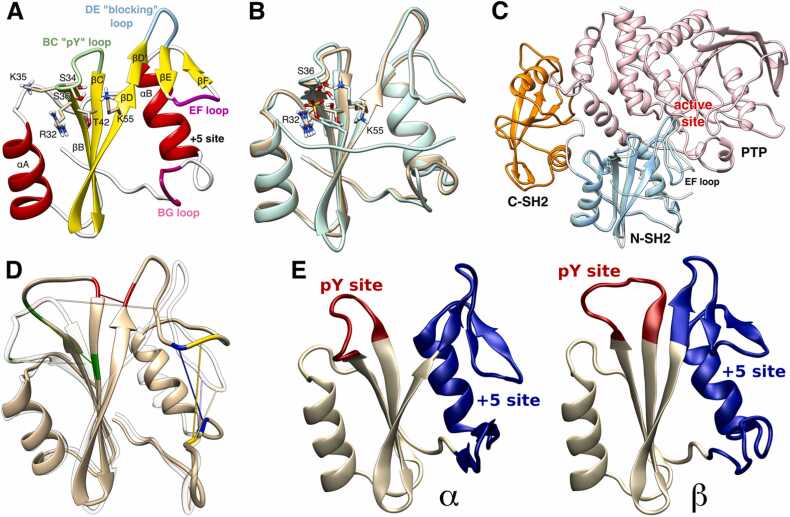
(A) Cartoon representation of the N-SH2 domain. Functionally important loops are highlighted in color: BC “pY” loop (green), DE “blocking” loop (light blue), EF loop (magenta), and BG loop (deep pink). The phosphotyrosine binds to the site delimited by the pY loop and the central β-sheet (βB, βC, βD strands). EF and BG loops delimit the + 5 site, where the peptide residue at position + 5 is hosted. (B) Overlay of the crystal structure of N-SH2 in apo form (*i.e.*, in the absence of a phosphopeptide, PDB ID 1AYD) with the crystal structure of N-SH2 complexed with a high-affinity phosphopeptide (PDB ID 1AYB). (C) Overlay of the crystal structure of autoinhibited SHP2 (PDB ID 4DGP) with the crystal structure of N-SH2 complexed with a phosphopeptide (PDB ID 1AYB). (D) Conformational transition from β (opaque) to α (transparent), representing the two main conformational states adopted by the N-SH2 domain, here visualized as the extreme projections onto the first PCA vector. The residues used to quantify the β-sheet spread, the pY loop opening, the binding cleft opening, and the + 5 site opening are highlighted in red, green, yellow, and blue, respectively. (E) Cartoon representation of the two conformations, α (left) and β (right), adopted by the N-SH2 domain, and shown as the extreme projections onto the first PCA vector. From the first PCA vector, two subvectors were selected, describing the conformations of the pY site (Ser^34^–Phe^41^, red cartoon) and of the + 5 site (Gln^57^–Glu^97^, blue cartoon), respectively. The residues used for defining the + 5 site include the end of the βD strand, the DE “blocking” loop, the EF loop, the αB helix, and the BG loop.

The structure of SHP2 consists of two Src homology 2 (SH2) domains arranged in tandem (N-SH2 and C-SH2), followed by the catalytic protein tyrosine phosphatase (PTP) domain, and a C-terminal tail that contains two key phosphorylation sites separated by a proline-rich motif [30], [31]. The SH2 domains are structurally conserved elements that recognize peptides containing a phosphorylated tyrosine (pY) [32]. Like other domains of the same family [32], N-SH2 consists of a central antiparallel β-sheet, composed of three β-strands, βB, βC, and βD, flanked by two α-helices, αA and αB (Fig. 1A). The peptide binds in an extended conformation perpendicular to the β-sheet [32]. A conserved “affinity pocket” lined by the BC loop (also called the phosphate-binding loop or pY loop) binds to pY-containing peptides 1000-fold more strongly than to their unphosphorylated counterparts [33]. Peptide residues C-terminal to the pY bind to a less conserved site flanked by the EF and BG loops that confers binding specificity (termed the “specificity pocket”) [32].

Under basal conditions, SHP2 adopts an autoinhibited state where the N-SH2 domain occludes the catalytic site of the PTP domain [34]. Phosphopeptide binding to N-SH2 weakens the autoinhibitory N-SH2–PTP interactions, leading to the release of N-SH2 from PTP and making the catalytic site accessible to substrates [35].

Due to its key role in SHP2 regulation, the structure of the isolated N-SH2 domain has been extensively studied. In 1994, Kuriyan and coworkers [36] solved the crystal structures of N-SH2, both unbound and bound to several high-affinity peptides, that were found to be similar to those of previously characterized SH2 domains [36]. However, the binding interface (or binding cleft) of N-SH2 extended to recognize up to the fifth residue after the phosphotyrosine (pY+5) [36], which was accommodated in a position of the specificity pocket termed the + 5 site. Notably, the structure of unliganded N-SH2 looked almost identical to those of its high-affinity complexes, except for some thermal fluctuations in the residues near the pY and BG loops (Fig. 1B) [36]. This suggested that N-SH2 does not undergo significant conformational changes upon peptide binding, raising the question of how peptide binding triggers the activation of SHP2. It was postulated that the activation of SHP2 probably involved conformational changes of other domains of SHP2, not just of N-SH2 [36].

In 1998, the first crystallographic structure of SHP2 at 2 Å resolution revealed a “closed” conformation, in which the N-SH2 domain interacts extensively with the PTP domain [37], and the DE loop of N-SH2 (termed the “blocking loop”) occludes the active site of PTP. Because of the inaccessibility of the active site, this conformation was described as autoinhibited. Comparison of the autoinhibited structure of SHP2 with the existing structures of the isolated N-SH2 domain showed that the EF loop and, to a lesser extent, the BG loop adopt significantly different conformations (Fig. 1C) [37], [38]. The proximity of the EF loop to the BG loop in the autoinhibited SHP2 conformation renders the N-SH2 domain unable to accommodate the C-terminal part of a phosphopeptide, contrary to the isolated N-SH2 domain, which exhibits an open binding cleft [37], [38]. Therefore, it became accepted that the closure of the N-SH2 binding cleft was due to the interaction with the PTP domain [37], [38]. Because peptide binding did not appear to be compatible with the conformation of N-SH2 in autoinhibited SHP2, it was proposed that the activation of SHP2 requires the spontaneous dissociation of the N-SH2 from PTP, followed by peptide binding and stabilization of the “open” active conformation [37], [38]. Thereafter, the opening of the binding cleft was considered the key feature driving SHP2 activation, in what became known as the “allosteric switch” model [37], [38].

However, another difference between the conformation of the N-SH2 domain in the autoinhibited structure of SHP2 and the liganded conformation of N-SH2 was initially ignored and consisted in the degree of zipping of the central β-sheet. This β-sheet was fully zipped in the autoinhibited structure of SHP2 and partially unzipped in the complexed N-SH2 domain. Perhaps, the apparent similarity of the liganded and unliganded conformations of isolated N-SH2, and their comparison with the autoinhibited structure of SHP2, initially led to overlook the possible role of the opening of the central β-sheet in the activation of SHP2.

The allosteric switch model has been recently challenged based on MD simulations and free energy calculations performed on the isolated N-SH2 domain and on autoinhibited SHP2, either in solution or in the crystal environment [39]. MD simulations in solution showed that the EF and BG loops are constitutively flexible and that SHP2 can retain its autoinhibited structure even with an open binding cleft [39]. Moreover, the binding cleft of N-SH2 in autoinhibited SHP2 was found to open up in solution, whereas the cleft remained closed in MD simulations of SHP2 in the crystal environment, likely due to crystal contacts [39]. Importantly, the opening of the binding cleft did not correlate with the free energy of activation of SHP2 [39], while the opening of the central β-sheet did [40]. Free energy calculations showed that the opening of the central β-sheet leads to the loss of surface complementarity between N-SH2 and PTP and thence to the activation of SHP2, while principal component analysis revealed an allosteric mechanism coupling the opening of the central β-sheet with the closure of both the pY loop and the + 5 site upon the phosphopeptide [40].

These allosteric effects were also observed in ^1^H,^15^N-HSQC spectra of N-SH2 in the presence of increasing concentrations of phosphotyrosine [41]. During the titration with this weak ligand, the largest chemical shift perturbations were observed in residues of the central β-sheet, the pY loop, the blocking loop, and the EF loop [41]. Importantly, the fact that NMR detected conformational changes upon phosphotyrosine binding challenged the hypothesis that unliganded and liganded N-SH2 share the same structure in solution.

The conformation of the unbound N-SH2 domain in the context of SHP2 activation was also recently revisited [42]. MD simulations of SHP2 in the absence of an activating ligand revealed that the binding cleft of N-SH2 was open in both the autoinhibited and active “open” conformations of SHP2, although the cleft was wider in the open state [42]. In these simulations, the central β-sheet retained a fully zipped, “closed” conformation, with limited fluctuation [42]. From these observations and the assumption that the conformation of unbound N-SH2 in open SHP2 is activating, the authors concluded that the activation of SHP2 is linked to the conformation of the loops and not of the central β-sheet of N-SH2 [42].

Given the ongoing debate on the precise structure of the unbound N-SH2 domain and the controversy surrounding its role in the activation of SHP2 [38], [39], [40], [41], [42], we adopted a multi-faceted approach to address some crucial questions about the structure of apo N-SH2 in solution. A critical revision of the available crystallographic data of the isolated N-SH2 domain was combined with an in-depth analysis of some key structural features in other SH2 domains, utilizing all available models resolved by X-ray diffraction and NMR. Next, we conducted molecular dynamics (MD) simulations of the isolated apo N-SH2 domain in solution, utilizing eight widely used force fields, and we compared the resulting MD simulation trajectories with experimental NMR residual dipolar couplings (RDCs) to derive the conformational ensemble that best represents the structure of apo N-SH2 in solution. We show that in its apo form, the isolated N-SH2 domain in solution predominantly adopts a conformation with a fully zipped, closed central β-sheet, and that N-SH2 undergoes widespread structural changes upon ligand binding, contradicting previous interpretations based on early crystallographic data. From these results, we conclude that the conformation of the isolated, apo N-SH2 domain is not predetermined to bind the phosphopeptide. Having previously also shown that the N-SH2 binding cleft is accessible to peptide binding in autoinhibited SHP2 [39], we infer that the pathway of activation of SHP2 does not necessarily involve the initial displacement of N-SH2 from PTP. Instead, our results, together with previous studies [39], [40], [41], suggest that early recognition of the pY moiety and, subsequently, of the peptide sequence by the N-SH2 domain in autoinhibited SHP2, triggers a series of conformational changes that lead to the unzipping of the β-sheet and the activation of SHP2.

## 2 Materials and methods

### 2.1 MD simulations of the apo N-SH2 domain in solution

The initial atomic coordinates were derived from the crystallographic structure of the N-SH2 domain in apo form (PDB ID 1AYD) [36]. The N-SH2 domain, consisting of residues 3–103, was placed at the center of a cubic box of edge length 7 nm, which was large enough to contain the domain and at least 1.5 nm of solvent on all sides. The protein was solvated with ∼10700 explicit water molecules. All MD simulations were performed with the GROMACS software package [43], using the force fields AMBER99SB [44], AMBER99SB*-ildnp [45], AMBER14SB [46], AMBER99SBws [47], AMBER03ws [47], CHARMM22* [48], CHARMM27 [49], and CHARMM36m [50]. The TIP3P [51] water model was used in combination with all force fields, with the exception of water-scaled (ws) AMBER force fields, for which the TIP4P/2005 [52] water model was used. Long-range electrostatic interactions were calculated with the particle-mesh Ewald (PME) approach [53]. A cutoff of 1.2 nm was applied to the direct-space Coulomb and Lennard-Jones interactions. Bond lengths and angles of water molecules were constrained with the SETTLE algorithm [54], and all other bonds were constrained with LINCS [55]. The solvent was relaxed by energy minimization, which was followed by 100 ps of MD at 300 K with harmonic restraints for the protein atoms. The system was then minimized without restraints and thermally equilibrated to 300 K over 10 ns in a stepwise manner. Starting from the last system structure, three independent replica exchange MD (REMD) simulations [56] of 1 ns each were spawned to equilibrate the respective 48 simulation replicas at temperatures ranging from 300 K to 400 K. The number of replicas ensured an estimated exchange probability of 0.12, and temperature values were determined using a temperature generator [57]. Initial velocities were generated from different random seeds at the corresponding temperatures according to the Maxwell-Boltzmann distribution. The pressure was set to 1 bar using the weak-coupling barostat [58]. After the equilibration, three independent, productive REMD simulations of 100 ns each were performed. The replica switches were attempted every 2 ps, and the average exchange probabilities between neighboring replicas ranged from 0.12 to 0.18. The pressures were set to 1 bar using the Parrinello-Rahman barostat [59]. The temperatures were controlled using velocity rescaling with a stochastic term [60].

### 2.2 MD simulations of the complexed N-SH2 domain in the crystal environment

The initial coordinates for the N-SH2 domain complexed with the IRS-1 pY895 peptide (SPGEpYVNIEFGS) were taken from a crystal structure (PDB ID 1AYB) [36]. This crystal belonged to the P4_3_2_1_2 space group and contained eight symmetry-related molecules. The tetragonal unit cell parameters were a = b = 6.29 nm, c = 7.78 nm, and α = β = γ = 90°. Missing or incomplete residues were modeled by Molecular Operative Environment (MOE) [61] using the AMBER12:EHT force field and enabling the periodic system of the P4_3_2_1_2 space group. The starting coordinates of the single-crystal unit cell were obtained by applying the P4_3_2_1_2 symmetry transformation. The AMBER99SB [44] force field, augmented with the parm99 dataset for phosphotyrosine [62], was used. The system was solvated with 6888 explicit water molecules [51] and 32 Na^+^ ions. The solvent box was generated by two successive solvent additions, each followed by a session of solvent relaxation, which comprised energy minimization followed by 100 ps MD at 300 K with harmonic restraints for the protein atoms. The system was then energy minimized without restraints and its temperature equilibrated in a stepwise manner to 300 K in 10 ns. Finally, a productive simulation of 500 ns was performed at constant volume.

### 2.3 Principal component analysis of the N-SH2 domain dynamics

Principal component analysis (PCA) was performed using the same vector **v**, reference structure, and procedure as reported in previous work [40], [63]. In short, from the PCA vector **v** for residues 6–101, subvectors were defined containing only the coordinates for residues Ser^34^–Phe^41^ and Gln^57^–Glu^97^. Technically, these subvectors were generated using PCA with only the respective subsets of atoms, applied to a trajectory which had been projected onto the full-length PCA vector **v**. The structures were superimposed by a least-squares fit on the backbone considering only the residues with smaller root mean-squared fluctuation, representing the relatively rigid core of the domain. The core was defined by the sequence ranges Phe^7^–Pro^33^, Asp^40^–Arg^47^, Ala^50^–Asn^58^, Asp^61^–Leu^65^, Phe^71^–Tyr^81^, Leu^88^–Glu^90^, Val^95^–Pro^101^.

### 2.4 Analysis of the crystal structure of the N-SH2 domain in apo form

The crystal coordinates, electron density map coefficients, and electron density difference map Fo–Fc of the N-SH2 domain in apo form were taken from the Research Collaboratory for Structural Bioinformatics (RCSB) Protein Data Bank (PDB ID 1AYD) [36]. Model building and refinement were carried out using Coot [64] version 0.8.8 and Refmac5 [65] (CCP4 [66] version 7.0.044). The original 1AYD structure and the map were validated by searching for unmodeled blobs of density. Two unexplained blobs of density (too big to be water molecules) were found. After removing water molecules 341 and 346, the second density blob was modeled with either a phosphate anion or with a sulfate anion. The electron density maps were updated after a cycle of refinement with Refmac5 and generated with the Fast Fourier Transform (FFT) tool of CCP4 suite.

### 2.5 Analysis of the SH2 domain structures

All atomic coordinates were taken from the RCSB Protein Data Bank. The list of the PDB entries including SH2 domains was taken from the PROSITE database (entry PS50001), which consisted of 481 hits as of December 2021 (see Supplementary Information) [67]. All biological assemblies of the PDB entries were considered, raising the number of hits up to 745 (see Supplementary Information). Each biological assembly was superposed over the structure of the *PTPN11* N-SH2 domain, previously used as a reference in the PCA. Structure superposition and sequence alignment were performed with MatchMaker of UCSF Chimera [68]: the best-aligning pair of chains between reference and target structure was performed using the Needleman-Wunsch alignment algorithm [69], considering a residue similarity term (substitution matrix BLOSUM-62 [70]), a secondary structure term (with score weight 30%), and gap penalties; secondary structure assignments were computed with DSSP [71]. Finally, the structural analyses (calculations of the principal components and distances) were performed on the superposed structures using GROMACS tools and the Bio3D package [72].

### 2.6 Sample preparation for NMR spectroscopy

The DNA encoding human N-SH2 (SHP2^1–105^) was cloned into pETM22 (European Molecular Biology Laboratory collection) and used to transform BL21(DE3) *E. coli*. The bacteria were grown at 37 °C in a shaker to an OD_600_ of 0.6–0.8, after which the culture was quickly cooled in an ice-water mix and induced with 0.2 mM IPTG at 20 °C. The bacteria were grown for 18 h, then harvested and the cell pellets were stored at –20 °C until purification. Bacterial growth was carried out in M9 minimal medium supplemented with kanamycin (50 μg/ml), ^15^NH_4_Cl (1 g/liter, Cambridge Isotope Laboratories), and ^13^C-D-glucose (4 g/liter, Cambridge Isotope Laboratories) to produce uniformly ^15^N,^13^C-labeled protein.

For protein purification, the bacterial pellets were resuspended in wash buffer (1 M NaCl, 50 mM tris (pH 7.6), 2% glycerol, 10 mM imidazole, and 5 mM β-mercaptoethanol) containing one tablet of EDTA-free protease inhibitor cocktail (Roche), 100 μg of lysozyme (Roth), and 50 μg of deoxyribonuclease (NEB). Lysis was performed by sonication, after which the lysate was clarified *via* centrifugation at 19000 rpm for one hour, and the supernatant was recovered and filtered, before loading it on a HisTrap HP column (GE Healthcare), previously equilibrated with wash buffer. The His_6_-tagged N-SH2 was eluted with a step gradient of 100% elution buffer (1 M NaCl, 50 mM tris (pH 7.6), 2% glycerol, 500 mM imidazole, and 5 mM β-mercaptoethanol). Cleavage of the thioredoxin tag was performed overnight at 4 °C with 3C protease, while excess imidazole was removed by dialysis against 2 liters of wash buffer. Purification proceeded with a second HisTrap step followed by size-exclusion chromatography on a HiLoad 16/600 Superdex 75 pg column (GE Healthcare), previously equilibrated with NMR buffer (100 mM MES (pH 6.8), 150 mM NaCl, 3 mM TCEP, 0.01% w/v sodium azide). Finally, the protein was concentrated to the desired value and either used directly or flash-frozen with liquid nitrogen for long-term storage at –80 °C.

### 2.7 NMR spectroscopy

NMR experiments for extraction of RDCs were collected on uniformly ^13^C,^15^N-labeled protein samples dissolved in NMR buffer (10 mM MES (pH 6.8), 150 mM NaCl, 5 mM DTT, 0.02% w/v sodium azide, 10% v/v D2O). Spectra were measured at a temperature of 298 K on an 850-MHz Bruker AVIII-HD spectrometer equipped with an inverse HCN CP-TCI cryogenic probe-head and running Bruker Topspin software (v3.2).

Each type of NMR experiment was recorded on isotropic and anisotropic (aligned) samples. The isotropic sample was prepared in a 3-mm diameter NMR tube, with a protein concentration of ∼500 μM and a sample volume of ∼200 μL. The anisotropic sample was prepared in a 5-mm diameter NMR tube, with a protein concentration of ∼200 μM and a sample volume of ∼500 μL, and contained Pf1 filamentous bacteriophage (ASLA Biotech, Latvia) at a concentration of ∼12.5 mg/ml. Initial inspection of the spectra of the anisotropic sample revealed that the degree of protein alignment was stronger than desired, so the NaCl concentration was increased from 150 mM to 250 mM to slightly attenuate the alignment. The formation and homogeneity of the anisotropic phase were confirmed by inspection of the ^2^H spectrum, which showed a well-resolved HOD doublet signal with a final splitting (after increasing the NaCl concentration) of 9.0 Hz.

Each type of RDC (H–N, N–C', H–C', C'–Cα, and Cα–Hα) was calculated as difference between the respective isotropic and anisotropic doublet-splittings. All doublet-splittings were extracted from IPAP-type (in-phase/anti-phase) spectra; in this approach, each RDC experiment comprises two sub-spectra (“in-phase” and “anti-phase”), in which the relevant doublet appears as either in-phase (the two doublet peaks have the same sign) or anti-phase (the two doublet peaks have opposite sign). Two new sub-spectra (“upfield” and “downfield”) that contain either one or other of the two doublet peaks were generated by taking the sum and difference of the in-phase and anti-phase sub-spectra. The peak-positions for calculation of the doublet-splittings were then measured from the upfield and downfield sub-spectra. H–N splittings were extracted from 2D IPAP–^15^N-HSQC spectra [73], and recorded with Hα/Hβ band-selective decoupling for ^15^N chemical-shift evolution [74]. C'–Cα splittings were extracted from 3D IPAP–HNCO[J-CA] spectra [75]. N–C' and H–C' splittings were both extracted from 2D IPAP-E.COSY–^15^N-HSQC spectra. In this experiment, the in-phase/anti-phase sub-spectra were generated by either refocusing or evolving ^15^N transverse magnetization with respect to the N–C' coupling prior to the indirect evolution period. The doublet components in the resultant 2D spectrum were separated by the N–C' splitting in the indirect dimension and by the H–C' splitting in the acquisition (direct) dimension. Cα–Hα splittings were extracted from 3D IPAP–HNCO(CA[J-HA]) spectra [76], where the splittings in the ^13^C indirect dimension derive from the Cα–Hα coupling but the chemical-shift positions of the signals correspond to those of the C' nuclei. The ^13^C chemical-shift evolution time (*t*_1,max_) was set to 21.4 ms, with the Cα–Hα coupling allowed to evolve for half that time (*t*_1,max_/2 = 10.7 ms). All spectra were processed with NMRPipe (v10.1) [77]. Peak-positions were determined with CcpNmr Analysis [78].

### 2.8 Back-calculation of RDCs from N-SH2 structures

RDCs were calculated after an alignment tensor best-fitting with the experimental RDCs using singular value decomposition (SVD). Calculations were performed using the calcTensor helper program of Xplor-NIH suite [79] on a maximum of 100 random configurations extracted from the simulations of N-SH2 in solution.

## 3 Results

### 3.1 Review of key interatomic distances in crystal structures of N-SH2

Experimental and theoretical approaches have revealed that the N-SH2 domain can adopt different conformations, both in solution and within a crystal [36], [37], [40], [41]. To define and differentiate these N-SH2 conformations, we have previously exploited two complementary strategies. The first strategy is to define specific interatomic distances, whose variations strongly correlate with known conformational transitions, while the second strategy is to define collective modes of motion using principal component analysis (PCA) [40].

We consider four key interatomic distances (Fig. 1D) for three crystal structures (Table 1, Fig. S1) that represent respectively the N-SH2 domain in inactive SHP2 (PDB ID 4DGP) [80], the isolated N-SH2 domain in complex with a phosphopeptide (PDB ID 1AYB) [36], and the isolated N-SH2 domain in the absence of a phosphopeptide (PDB ID 1AYD) [36]. The Lys^35^ Cα–Thr^42^ Cα distance describes the opening of the pY loop and, according to MD simulations in solution, this distance can range from ∼9 Å (closed pY loop) to ∼11 Å (open pY loop) (Fig. 1D, green lines) [40]. The Gly^39^ C–Asn^58^ N distance correlates with the spreading of the central β-sheet and it can range from ∼4 Å (closed β-sheet) to ∼12 Å (fully open β-sheet) (Fig. 1D, red lines) [40]. The Gly^67^ Cα–Lys^89^ Cα distance quantifies the closure of the binding cleft due to the displacement of the EF loop towards the BG loop, as observed in some crystal structures, and has been used as a reaction coordinate in previous calculations [39]. This distance can vary from ∼5 Å, when the binding cleft is completely inaccessible, to ∼14 Å, when the binding cleft is wide open (Fig. 1D, yellow lines) [39]. Finally, the Tyr^66^ Cα–Leu^88^ Cα distance correlates with the opening of the + 5 site, as determined by MD simulations in solution [40]. This distance integrates two structural changes: *i)* the vertical displacement of the EF loop towards the BG loop and *ii)* the horizontal displacement of the EF loop relative to the BG loop with the consequent torsion of the blocking loop. This distance varies from ∼7 Å, when the + 5 site is closed, to ∼12 Å, when the + 5 site is open (Fig. 1D, blue lines) [40].

**Table 1:** Reference interatomic distances (in Å) of crystal structures containing the N-SH2 domain.

	pY loop opening Lys^35^ Cα–Thr^42^ Cα	β-sheet spreading Gly^39^ C–Asn^58^ N	Binding cleft opening Gly^67^ Cα–Lys^89^ Cα	+ 5 site opening Tyr^66^ Cα–Leu^88^ Cα
**4DGP**	9.35	4.38	7.24	10.02
**1AYB**	8.71	7.34	12.53	12.00
**1AYD**	8.84	5.59	10.88	11.57

Despite the different liganded states of the N-SH2 domain in structures 4DGP, 1AYB, and 1AYD, the pY loop remains closed in all three structures (Lys^35^ Cα–Thr^42^ Cα distance range 8.7–9.3 Å). However, these structures differ in the spreading of the central β-sheet, which is closed in inactive SHP2 (4DGP, Gly^39^ C–Asn^58^ N distance 4.4 Å) but open in N-SH2 bound to a phosphopeptide (1AYB, 7.3 Å). Interestingly, the central β-sheet is partially open even in the isolated unliganded N-SH2 domain (1AYD), with the distance falling between the two previous cases (5.6 Å). Another distinguishing feature among these structures is the degree of opening of the binding cleft, which is almost completely closed in 4DGP (Gly^67^ Cα–Lys^89^ Cα distance 7.2 Å), but open in both liganded and unliganded isolated N-SH2 (12.5 Å and 10.9 Å for 1AYB and 1AYD, respectively). We have already shown that the closed binding cleft in the 4DGP structure is an artifact due to crystal contacts [39]. Finally, the Tyr^66^ Cα–Leu^88^ Cα distance describing the opening of the + 5 site is 11.6–12 Å in the isolated N-SH2 domain (1AYB, 1AYD), but it is 1.5–2 Å shorter in 4DGP because of the proximity of the EF and BG loops. This distance difference is significantly smaller than the variation observed in MD simulations because the lack of a horizontal component of the EF loop displacement does not permit the optimal alignment of the two reference atoms (*cf.* Fig. 1D and Fig. S1A). Therefore, in the 4DGP structure, the binding cleft is less accessible than suggested by the evaluation of the Tyr^66^ Cα–Leu^88^ Cα distance alone.

**Fig. 2:**
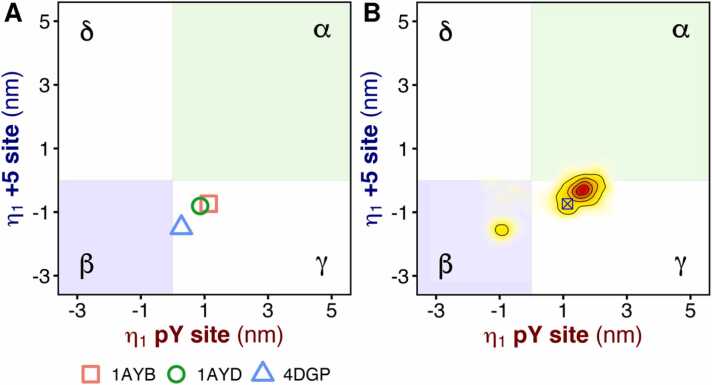
(A) Projection of three N-SH2 crystal structures onto the PCA subvectors of the pY site (*x*-axis) and + 5 site (*y*-axis). The structures represent the isolated N-SH2 complexed with the IRS-1 pY895 peptide (1AYB), the isolated N-SH2 in absence of a phosphopeptide (1AYD), and the N-SH2 in autoinhibited SHP2 (4DGP). The region corresponding to the α-state (pY loop closed, + 5 site closed) is shaded in green, while the region corresponding to the β-state (pY loop open, + 5 site open) is shaded in blue. The white regions correspond to the γ-state (pY loop closed, + 5 site open) and the *δ*-state (pY loop open, + 5 site closed). (B) Projection of the trajectory of N-SH2 complexed with the IRS-1 pY895 peptide in the crystal environment. For reference, the projection of the crystal structure is shown as a blue crossed square.

To address the potential ambiguity in describing complex collective motions using single interatomic distances, we used principal component analysis to describe the N-SH2 conformations (Fig. 1E) [40]. This allowed us to capture the essential features of a structure and characterize the conformations explored by the N-SH2 domain. Our previous work identified conformational states of N-SH2 in the essential plane spanned by two subvectors (*i.e.*, derived from the eigenvector corresponding to the first principal component of motion) that respectively describe the principal modes of the pY site and the + 5 site (red and blue cartoons in Fig. 1E, respectively) [40], [63]. The structures of N-SH2 were classified in terms of the conformations adopted by these two sites. The essential plane was divided into four conformational states (four shaded areas in Fig. 2A), corresponding to the pairwise combination of the open and closed states of both the pY and the + 5 sites. The α-state is characterized by *i*) a closed pY loop, *ii*) an increased distance between the ends of the two β-strands βC and βD, leading to the breakage of three inter-strand hydrogen bonds and the spreading of the central β-sheet into a Y-shaped structure, and *iii*) a closed + 5 site with a narrow, less accessible cleft, as shown in Fig. 1E [40]. In contrast, the β-state is characterized by *i*) an open pY loop, *ii*) a closed central β-sheet with fully zipped β-strands, and *iii*) an open + 5 site with an accessible cleft (Fig. 1E) [40]. The other two states correspond to a closed pY site with an open + 5 site (γ-state) and an open pY site with a closed + 5 site (*δ*-state) [40], [63]. Previous MD simulations suggested that the *β*-state of N-SH2 stabilizes the N-SH2–PTP contacts and, hence, the autoinhibited SHP2 conformation. In contrast, the *α*-state drives N-SH2 dissociation from PTP and SHP2 activation [39], [40].

The crystal structures of isolated N-SH2, either bound to a phosphopeptide (1AYB) or in the apo form (1AYD), are in the γ-state, with a closed pY site and an open + 5 site (Fig. 2A, Fig. S2), consistent with previous classifications based on interatomic distances. Interestingly, the conformation of the N-SH2 domain in SHP2 (4DGP crystal structure) is in between the γ- and the β-state (Fig. 2A). The pY site is partially closed but there is no spread of the central β-sheet, even though the pY loop is bent down towards the closed state. As we have previously shown, a bent, partially closed pY loop may result from the presence of an anion in the affinity pocket or from interactions with negatively charged residues of another protein replica [39], [40]. Interestingly, the + 5 site in 4DGP is classified as open, even though the binding cleft is closed, consistent with previous conclusions based on the comparison of the interatomic distances (Table 1). This result confirms that the closure of the binding cleft observed in some crystal structures may not fully project along the subvector describing the collective motion of the + 5 site detected by MD simulations in solution (Fig. S3). In addition, certain components of the collective motion of the + 5 site might be hindered in the crystal environment.

### 3.2 Crystal contacts in N-SH2 crystals influence the conformational distribution

Previously, our calculations have shown that the loops of N-SH2 are flexible and that their conformations may be easily affected by crystal contacts, with a resulting conformational bias [39]. In fact, in the crystal environment certain conformations may be either selected or precluded by the necessity of maintaining a given crystal packing or symmetry. More specifically in the case of N-SH2, our comparison of the available crystal structures has raised several questions. For example, is the transition between the different states of the + 5 site hindered by the crystal packing? Why are the structures of isolated N-SH2 so similar despite their different liganded states? And why is the pY loop nearly completely bent down towards the closed conformation in all crystal structures?

To address the first question, we performed MD simulations of the N-SH2 domain complexed with the IRS-1 pY895 peptide (corresponding to the complex of PDB ID 1AYB) in the crystal environment (Fig. S4), using the AMBER99SB force field. Previous simulations performed in solution with the same force field on N-SH2 bound to full-length phosphopeptides (including IRS-1 pY895) showed that the domain adopts either the β- or the α-state, depending on the peptide sequence and binding mode, and specifically on the orientation of the sidechain at position + 5 [40]. For this new set of simulations, the unit cell contained eight replicas of the N-SH2 domain, and all structures started in the γ-state. Based on the propensity for the β- and α-states observed in previous simulations in solution with the same force field, we would expect the structure to relax towards the β- or the α-state. However, only a small fraction of the sampled configurations switched to the β-state, whereas the vast majority continued to populate the γ-state (Fig. 2B). These results suggest that the crystal packing affects the conformation of the N-SH2 domain and restricts certain structural rearrangements, such as the opening and closing of the + 5 site. Notably, the structure adopted in 1AYB remains plausible, as the γ-state has been detected in some particular cases, for example in MD simulations performed in solution with the AMBER99SB force field on N-SH2 complexed with a truncated IRS-1 pY895 peptide lacking the residues in positions + 4 and + 5 [40], [63]. However, the interactions of the protein chain with other replicas in the crystal may perturb the structural ensemble and shift it towards the γ-state.

### 3.3 The electron density at the pY site of apo N-SH2 is compatible with a phosphate or sulfate ion

Irrespective of their liganded states, all crystal structures of the isolated N-SH2 domain feature an open central β-sheet, which is associated with the breakage of three inter-strand hydrogen bonds. The energetic penalty of this structural change is generally offset in the liganded form by the formation of a new interaction between the phosphopeptide and the pY loop upon closure of the pY site. This explains the closed pY site and the open central β-sheet observed in N-SH2 domains in complex with a phosphopeptide, but not in the unliganded form. However, the 1AYD structure of N-SH2 in the absence of a phosphopeptide suggests that the domain adopts an energetically suboptimal state, with a closed pY site and an open central β-sheet even in the absence of ligand. In this state, an energy penalty would arise from the broken hydrogen bonds of the β-sheet, as well as from the electrostatic repulsion of hydrogen bond donors in close proximity with each other in the closed pY loop.

**Fig. 3:**
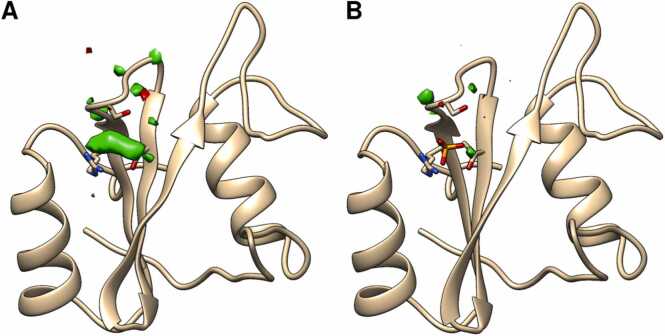
Electron density Fo-Fc difference map of the isolated N-SH2 domain in the absence (A) or presence (B) of a phosphate/sulfate anion at the affinity binding site. Structures and density maps were recalculated starting from the isolated N-SH2 domain (PDB ID 1AYD) after removing two water molecules placed at the pY site. Negative electron densities (excess electron density in the model relative to the experimental electron density) and positive electron densities (excess experimental electron density relative to that of the model) are colored in red and green, respectively. The isomesh represents the boundaries at ± 3σ, namely regions with a substantial level of disagreement. The N-SH2 domain is represented as cartoon. Anions and residues lining the affinity binding pocket are shown as sticks.

To resolve that paradox, we examined the experimental density map for unmodeled electron density. We found two sites of excess electron density, which are too large to be explained by the presence of water molecules. The first site is located near the EF loop and had been improperly modeled by two water molecules (residue IDs 363 and 364). However, this excess density is rather distant from the protein density and thus likely irrelevant. The second site of excess electron density, which had been also improperly modeled by a pair of water molecules (residue IDs 341 and 346), is located at the pY site, exactly where the phosphate group would be accommodated. Removal of the two water molecules reveals an extensive unmodeled excess electron density in the region surrounded by Arg^32^, Ser^34^, and Thr^42^ (Fig. 3A). Critically, this excess density is compatible with the presence of a phosphate or a sulfate anion in the pY site (Fig. 3B).

This finding suggests that 1AYB and 1AYD may show similar conformations because they both correspond to two liganded forms of N-SH2, the former in complex with the functional effector IRS-1 pY895 and the latter with an anion. Thus, based on these crystal structures, it is not possible to conclude whether the N-SH2 domain undergoes structural changes upon ligand binding.

### 3.4 Crystallography and NMR spectroscopy revealed systematically different SH2 conformations

**Fig. 4:**
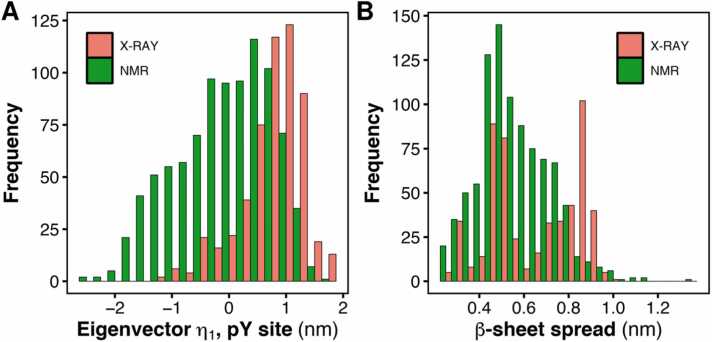
(A) Distribution of the projections onto the PCA subvector of the pY site and (B) distribution of the spreading of the central β-sheet for all structures containing at least one SH2 domain (PROSITE entry PS50001) that share high homology with *PTPN11* N-SH2. The complete set of structures was divided into two subsets according to the experimental methodology: X-ray diffraction (red bars) or NMR spectroscopy (green bars).

To further evaluate the effect of crystal packing on the flexible loops of N-SH2, we analyzed a set of deposited structures containing at least one SH2 domain, both liganded and unliganded (PROSITE entry PS50001). We focused on the most conserved region, the pY site, and on SH2 domains bearing structural similarity with N-SH2. Starting from 481 PDB entries, we considered their biological assemblies, leading to a final set of 745 entries (see Supplementary Information). Of the 745 entries, 558 were used for the analysis, whereas 187 were discarded either because the length of the pY loop was different from that of N-SH2 (eight-residue-long pY loop in N-SH2, Ser^34^–Phe^41^) or because the backbone was not completely resolved. Considering all models in the entries, 1472 conformations were analyzed, and the projection along the subvector that describes the opening/closing of the pY site was calculated for each conformation (Fig. 4A). The distribution of projections showed a significant variance, indicating that the vector identified from the PCA of MD simulations was a sensitive and accurate descriptor of the structural rearrangement of the pY site in the large set of analyzed PDB structures. However, grouping the projections according to the respective experimental method revealed different distributions: the structures resolved by X-ray diffraction showed a clear preference for positive values of the projection, corresponding to a predominantly closed pY site, whereas the NMR structures showed a bimodal distribution with positive and negative projections, indicating that the pY site adopted both closed and open conformations. Only a few crystal structures reported a fully open pY site (*e.g.*, 2B3O structure of SHP1, see Fig. S5), while some NMR ensembles covered the complete transition from a closed to an open pY site (*e.g.*, 1AB2 structure of c-ABL SH2 domain, see Fig. S6).

Similarly, the distributions of the distance representing the spreading of the central β-sheet were different depending on the experimental techniques (Fig. 4B): X-ray diffraction structures revealed a distribution with three maxima corresponding respectively to a closed, a partially open, and a fully open β-sheet (Fig. 4B, red), while NMR structures showed a continuous distribution with a predominance of closed β-sheet (Fig. 4B, green).

Not all deposited structures containing SH2 domains adhere to the linear correlation between the opening of the pY site and the spreading of the central β-sheet observed for N-SH2 in solution (*cf.* Fig. 4A and 4B). It is safe to assume that, first, not all SH2 domains have the same correlated internal motions as N-SH2 and, second, the crystal environment can perturb these correlated motions.

From this analysis, which shows a strong predominance of closed pY loop conformations in structures determined by X-ray crystallography, as compared to those determined by NMR spectroscopy, we conclude that the lack of conformational variability observed for the pY loop in the crystal structures is likely caused by crystal packing and in general by non-physiological experimental conditions.

### 3.5 The MD ensembles of apo N-SH2 are dependent on the protein force field

We have shown that the crystallographic environment can significantly influence the structure of the isolated N-SH2 domain. For this reason, we used, as an orthogonal method to X-ray crystallography, a combination of NMR spectroscopy and MD simulations to determine the structure of unliganded isolated N-SH2 in solution.

First, we performed replica-exchange MD simulations using eight commonly used force fields: AMBER99SB, AMBER99SB*-ildnp, AMBER14SB, AMBER99SBws, AMBER03ws, CHARMM27, CHARMM22*, and CHARMM36m [44], [45], [46], [47], [48], [49], [50]. We provide a summary of the parameterization history and key features of each force field in the Supplementary Information. Our objectives in using multiple force fields were twofold: *i)* to discover and characterize possible differences among the respective ensembles and *ii)* to determine which force field best reproduces the experimental NMR residual dipolar couplings of the unliganded isolated N-SH2 domain.

Fig. S7 displays the root mean-square fluctuations (RMSFs) of the Cα atoms in the N-SH2 domain, determined for each of the eight force fields. The profiles of the RMSF values are similar; however, the AMBER03ws force field displayed larger fluctuations, particularly in the region corresponding to the BG loop.

To assess the overall heterogeneity of the conformational ensembles obtained with the eight force fields, we carried out a cluster analysis. The structural variability of the + 5 site and the pY site are key factors contributing to the heterogeneity of an ensemble. Fig. S8 shows the empirical cumulative distributions of the N-SH2 conformations as a function of the number of most populated clusters. The AMBER99SB force field yielded a large first cluster that encompassed almost one-quarter of all conformations, indicating that the generated ensemble exhibited moderate structural variability. The AMBER03ws force field produced the most structurally diverse ensemble. All other force fields were between these two extreme cases and yielded similar distributions.

To quantitatively characterize the structural ensembles, we used the principal components and the reference interatomic distances that we previously defined and that quantify respectively the *β*-sheet spread, the pY loop opening, and the + 5 site opening.

**Fig. 5:**
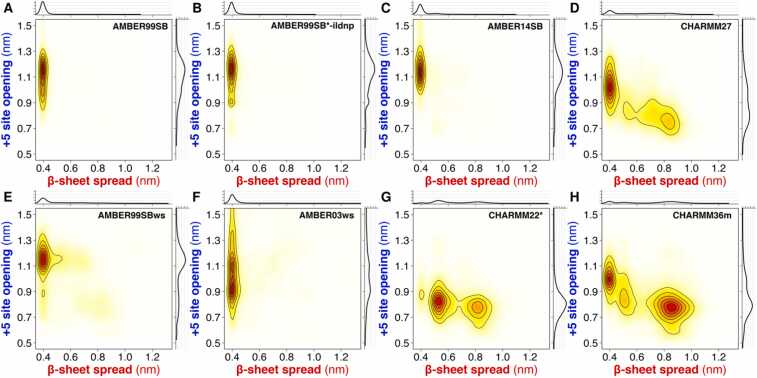
Bivariate probability distributions with respect to the β-sheet spread and the + 5 site opening for each force field (see panel titles). Univariate distributions with respect to the β-sheet spread and the + 5 site opening are shown as axis projections along the respective axes.

Fig. 5 presents the conformational ensembles projected onto the pair of interatomic distances characterizing the β-sheet spread and the + 5 site opening. Because the opening of the pY loop is largely coupled to the spreading of the central β-sheet in each ensemble (Fig. S9), we can describe the conformations of the N-SH2 domain in solution by using only two interatomic distances. The AMBER force fields predominantly yielded a closed central β-sheet and an open + 5 site. However, AMBER99SB and AMBER99SB*-ildnp strongly stabilized the central β-sheet, as evidenced by the peaked distribution along the β-sheet spread (Fig. 5A,B). On the other hand, other AMBER variants, such as AMBER99SBws or AMBER03ws, allowed for a small fraction of a partially open β-sheet (Fig. 5E,F). Notably, AMBER03ws yielded numerous outliers with an excessively open + 5 site (distance ≫ 14 Å), characterized by a structurally disordered BG loop (Fig. 5F).

Compared to the AMBER variants, the CHARMM force fields yielded significantly different ensembles with greater structural heterogeneity. Specifically, CHARMM27 predominantly generated a closed central β-sheet, although a significant fraction of the conformations adopted an open central β-sheet (Fig. 5D). Conversely, CHARMM36m generated a highly heterogeneous ensemble with similar populations of open and closed central β-sheets, while maintaining a mostly closed + 5 site (Fig. 5H). A different heterogeneous ensemble was generated using CHARMM22*, for which the majority of the central β-sheets adopted a partially open conformation, primarily driven by backbone–side-chain interactions (*e.g.*, with Asn^58^) replacing backbone–backbone interactions between the two β-strands (Fig. 5G).

The probability distributions of the conformational states of the N-SH2 domain confirmed these results. All the AMBER force fields yielded N-SH2 in the β-state (Fig. S10). However, the CHARMM force fields gave less univocal results (Fig. S11): CHARMM27 and CHARMM36m favored the β- and the α-state of N-SH2, respectively, while CHARMM22* yielded intermediate conformations between these two states.

### 3.6 Comparison of MD ensembles with residual dipolar couplings

**Fig. 6:**
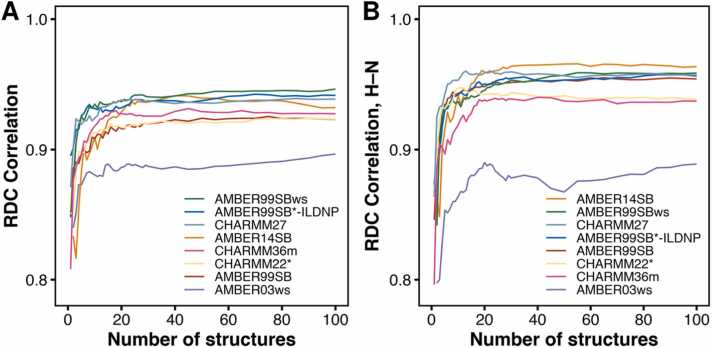
Pearson correlation coefficient between the experimental and back-calculated RDCs *versus* the number of structures belonging to the ensemble for each force field. The RDCs were calculated using an unweighted set of structures randomly selected from each ensemble and one alignment tensor. The correlation coefficients were calculated after fitting over all experimental RDCs and reported (A) for the whole set of RDCs or (B) only for the H–N bond-RDCs.

To unambiguously determine the conformational state of the isolated apo N-SH2 domain in solution, we measured NMR residual dipolar couplings (RDCs). We analyzed the agreement between this set of experimental RDCs and the corresponding sets of back-calculated RDCs derived from the structural ensemble of each force field. The alignment tensor was calculated from the experimental RDCs using singular value decomposition (SVD) [81], with all members of the structural ensemble fitted simultaneously to the experimental RDCs in such a way as to yield a single alignment tensor and an ensemble-averaged set of back-calculated RDCs. For each force field, we considered an unweighted set of up to 100 structures randomly selected from the corresponding structural ensemble (Fig. 6A). All force fields, except for AMBER03ws, exhibited a strong correlation, greater than 0.9. The highest correlation between back-calculated and experimental RDCs was obtained with AMBER99SBws, AMBER99SB*-ildnp and CHARMM27. Amongst the CHARMM force fields, CHARMM27 exhibited a higher correlation than CHARMM36m or CHARMM22*.

To investigate the origin of these subtle differences between the force fields, we focused on the correlations of the H–N bond-RDCs due to the higher relative precision associated with their measurements (Table S1). Also for this subset of RDCs, most of the AMBER force fields and CHARMM27 provided the highest correlations (Fig. 6B). We compared the experimental H–N RDCs with the back-calculated RDCs for each force field, with the panels in Fig. S12 ordered according to decreasing Pearson correlation coefficient *R*. RDCs representing outliers were labeled with the corresponding residue number (Fig. S13). The unstructured flexible residues Gly^39^, which delimits the β-strand βD, and Asp^94^, which is situated at the apical position of the BG loop, were outliers for almost all force fields. With a few exceptions, AMBER14SB, AMBER99SBws, AMBER99SB*-ildnp, and CHARMM27 provided an excellent correlation, especially for all structured residues (*i.e.*, residues in an α-helix or in β-strand). In contrast, AMBER03ws yielded the poorest correlation with several outliers clustering in the region of the BG loop (*e.g.*, Gln^87^, Lys^89^, Lys^91^). Given that AMBER03ws exhibited the greatest structural disorder at the + 5 site and produced the worst correlation to the RDCs, with many outliers at the BG loop, we infer that the flexibility of the + 5 site in apo N-SH2 is limited to that displayed by the AMBER14SB, AMBER99SBws, AMBER99SB*-ildnp, and CHARMM27 force fields. Finally, CHARMM36m and CHARMM22* yielded significantly worse correlations with many outliers in the EF loop (*e.g.*, Gly^67^, Gly^68^, Glu^69^). Given that CHARMM36m and CHARMM22* were the only force fields that assigned the α-state (with an open central β-sheet) to N-SH2 and that these force fields yielded poorer correlations (particularly in the region of the EF loop involved in the transition between the α- and the β-state), we infer that the α-state is not significantly populated in solution. Collectively, based on the comparison with experimental RDCs, we conclude that the isolated, apo N-SH2 domain in solution primarily adopts the β-state and thus possesses a closed central β-sheet.

## 4 Discussion

Over the last few decades, the conformation of the unbound N-SH2 domain and its role in the activation mechanism of full-length SHP2 have been extensively discussed. The assumption, based on early crystallographic data, that the liganded and unliganded conformations of isolated N-SH2 are similar, and thus the conformation of unliganded N-SH2 is equivalent to the conformation of N-SH2 in activated SHP2, has supported the theory that SHP2 activation invariably occurs through the fraction of basally active, open SHP2. Under this assumption, the binding of a phosphopeptide merely serves to stabilize the activating conformation of N-SH2 in open SHP2. This theory was further endorsed by the observation that the binding cleft, accommodating activating phosphopeptides, is not accessible in the crystallographic structure of autoinhibited SHP2, except for the pY site itself [37].

**Fig. 7:**
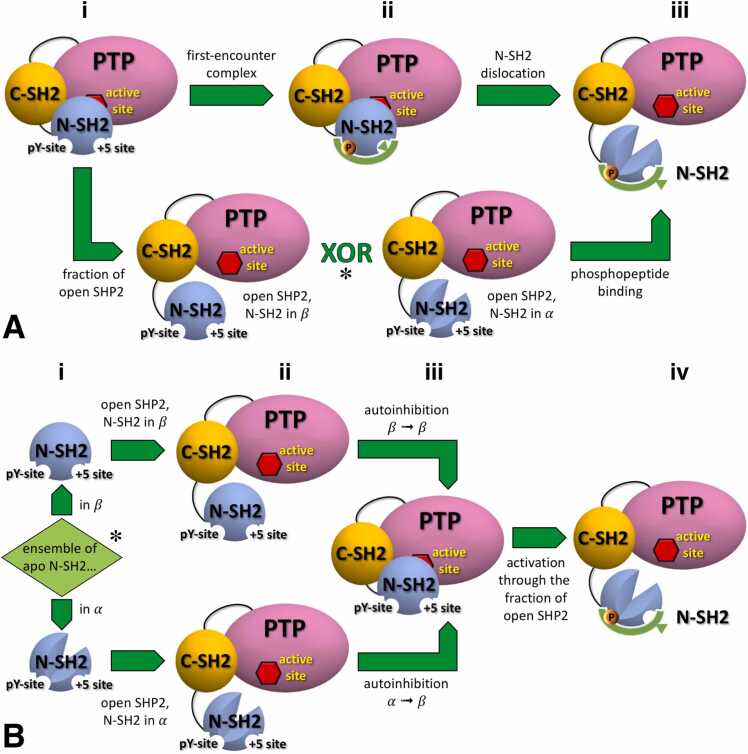
This simplified scheme illustrates (A) the two alternative mechanisms of SHP2 activation (sublabels from i to iii): either the formation of a first-encounter complex between the peptide and closed SHP2 or a transition through the fraction of basally active, open SHP2 (ii). In the case of a transition through the fraction of open SHP2 (see asterisk *), the scheme illustrates (B) the two alternative scenarios based on the conformation adopted by the isolated N-SH2 in its apo form (sublabels from i to iv). In its unliganded form, the ensemble of the isolated N-SH2 domain can predominantly populate one of two possible conformations (i), labeled as α (lower panel) and β (upper panel). By definition, the α conformation activates SHP2, while the β conformation stabilizes autoinhibited SHP2. In open SHP2 in the absence of phosphopeptide binding, the N-SH2 domain adopts the same conformation as observed in the corresponding apo form of the isolated domain, given that no specific interactions with the PTP domain are present (ii). Regardless of the conformation adopted by the apo form, N-SH2 adopts the stabilizing β conformation in autoinhibited SHP2 (iii) and the activating α conformation under stimulating conditions when a phosphopeptide binds to N-SH2 (iv).

In our previous study [39], we demonstrated that the N-SH2 binding cleft is accessible in autoinhibited SHP2 in solution. Therefore, the transition through the fraction of basally active, open SHP2 is not mandatory for activation. Indeed, the accessibility of the N-SH2 binding cleft in autoinhibited SHP2 allows another mechanism of activation which involves the formation of a first-encounter complex between the peptide and closed SHP2, followed by reshaping of the N-SH2 domain, loss of complementarity between N-SH2 and PTP, and, finally, dislocation of the N-SH2 domain (Fig. 7A).

In the case of SHP2 activation through the fraction of open SHP2, the influence of the conformation of unbound N-SH2 is particularly subtle (Fig. 7A). In fact, the unbound N-SH2 domain can, in principle, adopt at least two conformations: one capable of activating SHP2 (generically denoted here as α) and one that stabilizes its inactive state (generically denoted here as β). It is important to note that the existence of an activating (α) and a stabilizing (β) conformation can be assumed independently of the knowledge of the structural elements that truly cause the activation, even though, in agreement with our findings, the opening of the central β-sheet, and not of the binding cleft, is shown as the activating feature in Fig. 7. By definition, the stabilizing β conformation is adopted in autoinhibited SHP2, whereas the activating α conformation is populated when SHP2 is activated in the presence of a phosphopeptide binding to N-SH2.

Although we could consider an ensemble of unbound N-SH2 as a mixture of α and β conformations, for simplicity we consider the two extreme cases, where unbound N-SH2 adopts nearly completely either the α or β conformations (Fig. 7 Bi, *cf.* Fig. 7Aii). This assumption, despite being a simplification, does not alter the overarching conclusion of this discussion: the activation pathway requiring transition through the fraction of basally active SHP2 remains plausible irrespective of the specific conformation of unbound N-SH2.

Indeed, each alternative ensemble of the unbound N-SH2 domain in open SHP2 (Fig. 7 Bii, *cf.* Fig. 7Aii) would adopt the same conformation as observed in its corresponding apo form of the isolated domain (Fig. 7 Bi). This is due to the absence of specific interactions with the PTP domain that might lead to a perturbation of the ensemble.

In autoinhibited SHP2 the N-SH2 domain inherently adopts the stabilizing β conformation (Fig. 7 Biii). Therefore, in the hypothetical scenario where the unbound N-SH2 domain adopts the β conformation (Fig. 7 Bii), binding to PTP and forming autoinhibited SHP2 would require no significant conformational changes, following a lock-and-key model. In this case, the finite binding affinity of unbound N-SH2 in the β conformation to PTP implies that even under non-stimulating conditions (absence of phosphopeptides), the open state of SHP2 is partially populated. Conversely, if the unbound N-SH2 domain adopted the activating α conformation (Fig. 7Bii), a conformational transition would be necessary before N-SH2 can bind to PTP, resembling an induced fit model. The extent of open SHP2 depends on the free energy required to switch the N-SH2 conformation from α to β. The larger this free energy, the greater the fraction of open SHP2 under non-stimulating conditions.

Furthermore, when considering the binding of a phosphopeptide to open SHP2 (Fig. 7 Biv), two scenarios are possible. If the unbound N-SH2 domain is in the stabilizing β conformation (Fig. 7 Bii), phosphopeptide binding induces a conformational transition to the α conformation. Conversely, if the N-SH2 domain is in the activating α conformation (Fig. 7 Bii), the phosphopeptide simply stabilizes N-SH2 in this conformation.

In conclusion, irrespective of the conformation, α or β, adopted by the unbound N-SH2, we would obtain two alternative plausible scenarios, each characterized by its own thermodynamics and kinetics. If unbound N-SH2 were in the stabilizing β conformation, the level of SHP2 basal activity would be the lowest, and phosphopeptide binding would require surpassing a conformational energy barrier, offering a higher level of control of SHP2 stimulation. If unbound N-SH2 were in the activating α conformation, activation of SHP2 would likely be less selective.

## 5 Conclusion

Using a combination of MD simulations and NMR residual dipolar couplings (RDCs), we found that the isolated apo N-SH2 domain in solution adopts a conformation similar to that of PTP-bound N-SH2, with a closed, fully zipped, central β-sheet, but different to the conformation of phosphopeptide-bound N-SH2. Thus, our data supports the first scenario described above, which implies low basal SHP2 activity while providing a high level of control of SHP2 stimulation. This finding is consistent with previous observations that unzipping of the β-sheet can be initiated by the binding of weak ligands (*e.g.*, during titration of the N-SH2 with an increasing concentration of phosphotyrosine) [41]. We also found that the crystal environment can hamper certain conformational transitions and affect the conformations of flexible loops through interactions with other protein replicas or crystallization agents and precipitants, which explains the discrepancy between the conformations of the unliganded isolated N-SH2 in solution and in the crystal. In particular, we discovered that the crystallographic structure of the apo N-SH2 domain (PDB ID 1AYD) was biased by the binding of a ligand, likely a sulfate anion [36], at the pY site.

Certain AMBER force fields, such as AMBER99SBws and AMBER99SB*-ildnp, and the CHARMM27 force field provided the best agreement with the experimental RDCs. On the other hand, the AMBER99SB force field tended to over-stabilize the central β-sheet, while the AMBER03ws force field generated an excessively disordered + 5 site. Critical comparison of the ensembles revealed that the central β-sheet is slightly less stable than previously implied by simulations with the AMBER99SB force field [40], [41]. Additionally, the EF and BG loops lining the + 5 site are flexible but not overly disordered, which means that an excessively open binding cleft [42] is incompatible with the experimental RDCs.

In conclusion, in this work we have resolved a long-standing question around the structure of the unliganded N-SH2 domain of SHP2, providing a coherent structural basis for the tightly regulated activation of SHP2. We have also demonstrated the need to determine structures in solution, uncovering some important pitfalls of crystallographic structures of small, allosterically regulated domains.

## Author contributions

M.M. prepared samples. M.M. and J.K. recorded and analyzed NMR data. T.C. designed and supervised NMR experiments. M.A. conceived the research. M.A. designed, supervised and performed MD simulations, structural analysis and comparison with experimental data. M.A. drafted the manuscript. All authors reviewed and edited the final version of the manuscript.

## Declaration of Competing Interest

The authors declare that they have no known competing financial interests or personal relationships that could have appeared to influence the work reported in this paper.
