# Supplementary material for: Phosphopeptide binding to the N-SH2 domain of tyrosine phosphatase SHP2 correlates with the unzipping of its central β-sheet
Source: Comput Struct Biotechnol J. 2024 Mar 2;23:1169–80. doi: 10.1016/j.csbj.2024.02.023 (PMC10951427; doi:10.1016/j.csbj.2024.02.023)
Supplement: Supplementary file 1 — Supplementary material [file mmc1.docx]

**Supplementary Information**

**Phosphopeptide binding to the N-SH2 domain of tyrosine phosphatase SHP2 correlates with the unzipping of the central β-sheet**

Michelangelo Marasco,^1^ John Kirkpatrick,^2^ Teresa Carlomagno,^2,3^ Jochen S. Hub,^4^ Massimiliano Anselmi^4,^*

^1^Molecular Pharmacology Program, Memorial Sloan Kettering Cancer Center, New York, NY, USA

^2^School of Biosciences, University of Birmingham, Edgbaston, B15 2TT, Birmingham, UK

^3^Institute of Cancer and Genomic Sciences, University of Birmingham, Edgbaston, B15 2TT, Birmingham, UK

^4^Theoretical Physics and Center for Biophysics, Saarland University, 66123 Saarbrücken, Germany

* To whom correspondence may be addressed:

E-mail: massimiliano.anselmi@uni-saarland.de (M.A.)

**Table S1. H–N Splittings and RDCs.** Doublet splittings for backbone H–N groups measured under isotropic conditions ($\Delta_{\text{HN}}^{\text{iso}}$; $\sigma\left[ \Delta_{\text{HN}}^{\text{iso}} \right]$ denotes the estimated experimental uncertainties) and anisotropic conditions (phage-aligned, $\Delta_{\text{HN}}^{\text{aniso}}$; uncertainties: $\sigma\left[ \Delta_{\text{HN}}^{\text{iso}} \right]$), and corresponding RDCs ($D_{\mathrm{HN}}$; uncertainties: $\sigma\left[ D_{\mathrm{HN}} \right]$). All values are given in units of Hz. Experimental uncertainties were estimated based on an empirical equation relating the uncertainty of the position of a peak ($\sigma\left[ \nu\right]$, in Hz) to its linewidth (LW, also in Hz) and its signal-to-noise (S/N = peak-height/root-mean-square noise): $\sigma\left[ \nu\right]\sim0.5\times\left( \frac{\text{LW}}{\text{S/N}} \right)$.

| Residue | $\boldsymbol{\Delta}_{\text{HN}}^{\text{iso}}$ | $\boldsymbol{\sigma}\left[ \boldsymbol{\Delta}_{\text{HN}}^{\text{iso}} \right]$ | $\boldsymbol{\Delta}_{\text{HN}}^{\text{aniso}}$ | $\boldsymbol{\sigma}\left[ \boldsymbol{\Delta}_{\text{HN}}^{\text{iso}} \right]$ | $\boldsymbol{D}_{\mathbf{HN}}$ | $\boldsymbol{\sigma}\left[ \boldsymbol{D}_{\mathbf{HN}} \right]$ |
| --- | --- | --- | --- | --- | --- | --- |
| Trp6 | –90.71 | 0.025 | –69.99 | 0.093 | 20.72 | 0.097 |
| Phe7 | –93.05 | 0.031 | –115.28 | 0.204 | –22.23 | 0.207 |
| His8 | –93.35 | 0.032 | –112.62 | 0.202 | –19.27 | 0.204 |
| Asn10 | –91.96 | 0.016 | –104.30 | 0.062 | –12.35 | 0.064 |
| Ile11 | –92.87 | 0.022 | –79.56 | 0.057 | 13.32 | 0.061 |
| Thr12 | –93.23 | 0.024 | –78.87 | 0.143 | 14.36 | 0.145 |
| Gly13 | –93.20 | 0.120 | –106.80 | 0.163 | –13.60 | 0.404 |
| Val14 | –92.56 | 0.011 | –97.74 | 0.081 | –5.18 | 0.081 |
| Glu15 | –93.24 | 0.014 | –88.04 | 0.085 | 5.21 | 0.087 |
| Ala16 | –93.97 | 0.013 | –104.98 | 0.072 | –11.01 | 0.073 |
| Glu17 | –93.34 | 0.012 | –105.84 | 0.072 | –12.50 | 0.073 |
| Asn18 | –94.54 | 0.013 | –98.50 | 0.078 | –3.96 | 0.158 |
| Leu19 | –93.82 | 0.014 | –95.06 | 0.084 | –1.24 | 0.086 |
| Leu20 | –93.90 | 0.017 | –109.87 | 0.119 | –15.97 | 0.120 |
| Leu21 | –93.11 | 0.016 | –101.00 | 0.083 | –7.89 | 0.170 |
| Thr22 | –91.92 | 0.015 | –83.28 | 0.076 | 8.64 | 0.077 |
| Arg23 | –91.42 | 0.015 | –99.73 | 0.128 | –8.30 | 0.129 |
| Gly24 | –94.08 | 0.015 | –108.82 | 0.065 | –14.74 | 0.067 |
| Val25 | –91.35 | 0.023 | –67.60 | 0.077 | 23.75 | 0.161 |
| Asp26 | –93.97 | 0.015 | –81.04 | 0.130 | 12.93 | 0.131 |
| Gly27 | –93.04 | 0.027 | –116.67 | 2.378 | –23.64 | 2.379 |
| Ser28 | –94.17 | 0.022 | –72.67 | 0.098 | 21.50 | 0.101 |
| Phe29 | –93.53 | 0.040 | –119.52 | 0.231 | –26.00 | 0.469 |
| Leu30 | –92.96 | 0.021 | –121.86 | 0.180 | –28.90 | 0.181 |
| Ala31 | –92.71 | 0.033 | –117.59 | 0.290 | –24.88 | 0.292 |
| Arg32 | –92.89 | 0.025 | –107.51 | 0.128 | –14.62 | 0.130 |
| Ser34 | –92.80 | 0.016 | –67.63 | 0.195 | 25.17 | 0.391 |
| Asn37 | –92.42 | 0.111 | –103.34 | 0.143 | –10.92 | 0.181 |
| Gly39 | –93.33 | 0.038 | –96.09 | 0.064 | –2.75 | 0.074 |
| Asp40 | –92.83 | 0.013 | –89.85 | 0.036 | 2.98 | 0.038 |
| Phe41 | –92.85 | 0.027 | –86.36 | 0.135 | 6.49 | 0.138 |
| Thr42 | –92.93 | 0.025 | –105.22 | 0.128 | –12.29 | 0.131 |
| Leu43 | –92.52 | 0.031 | –113.76 | 0.189 | –21.24 | 0.192 |
| Ser44 | –93.52 | 0.026 | –118.70 | 0.309 | –25.18 | 0.310 |
| Val45 | –92.95 | 0.020 | –125.08 | 0.250 | –32.13 | 0.501 |
| Arg46 | –93.75 | 0.022 | –115.58 | 0.178 | –21.83 | 0.179 |
| Arg47 | –93.30 | 0.024 | –97.53 | 0.090 | –4.23 | 0.093 |
| Asn48 | –93.71 | 0.024 | –104.17 | 0.113 | –10.46 | 0.116 |
| Gly49 | –92.94 | 0.021 | –108.75 | 0.087 | –15.81 | 0.090 |
| Ala50 | –92.78 | 0.011 | –77.64 | 0.056 | 15.14 | 0.057 |
| Val51 | –92.27 | 0.013 | –91.47 | 0.040 | 0.80 | 0.042 |
| Thr52 | –92.67 | 0.020 | –117.87 | 0.194 | –25.19 | 0.195 |
| His53 | –93.52 | 0.024 | –114.92 | 0.168 | –21.41 | 0.339 |
| Ile54 | –92.45 | 0.024 | –119.92 | 0.334 | –27.47 | 0.335 |
| Lys55 | –93.42 | 0.018 | –111.58 | 0.081 | –18.16 | 0.083 |
| Ile56 | –92.67 | 0.039 | –82.21 | 0.171 | 10.47 | 0.176 |
| Gln57 | –93.08 | 0.043 | –88.48 | 0.201 | 4.60 | 0.205 |
| Asn58 | –92.64 | 0.033 | –93.21 | 0.114 | –0.58 | 0.118 |
| Thr59 | –92.73 | 0.050 | –103.63 | 0.128 | –10.90 | 0.274 |
| Gly60 | –94.17 | 0.029 | –90.57 | 0.055 | 3.61 | 0.062 |
| Asp61 | –91.84 | 0.033 | –100.52 | 0.112 | –8.68 | 0.117 |
| Tyr62 | –92.54 | 0.026 | –109.37 | 0.122 | –16.83 | 0.124 |
| Tyr63 | –91.21 | 0.060 | –103.65 | 0.287 | –12.44 | 0.293 |
| Asp64 | –94.38 | 0.028 | –91.09 | 0.114 | 3.29 | 0.117 |
| Leu65 | –91.54 | 0.078 | –80.70 | 0.323 | 10.84 | 0.332 |
| Tyr66 | –92.17 | 0.068 | –90.93 | 0.791 | 1.24 | 0.794 |
| Gly67 | –92.31 | 0.207 | –103.57 | 0.533 | –11.27 | 0.572 |
| Gly68 | –94.29 | 0.062 | –96.45 | 0.183 | –2.17 | 0.193 |
| Glu69 | –92.87 | 0.021 | –82.15 | 0.099 | 10.71 | 0.101 |
| Lys70 | –92.40 | 0.036 | –74.88 | 0.089 | 17.52 | 0.096 |
| Phe71 | –92.25 | 0.031 | –91.65 | 0.161 | 0.61 | 0.164 |
| Ala72 | –92.32 | 0.021 | –98.27 | 0.089 | –5.95 | 0.092 |
| Thr73 | –92.07 | 0.021 | –89.28 | 0.072 | 2.78 | 0.075 |
| Leu74 | –93.16 | 0.026 | –91.08 | 0.172 | 2.08 | 0.174 |
| Ala75 | –92.96 | 0.014 | –87.15 | 0.098 | 5.82 | 0.099 |
| Glu76 | –93.52 | 0.017 | –89.29 | 0.070 | 4.23 | 0.072 |
| Leu77 | –93.05 | 0.019 | –88.70 | 0.129 | 4.35 | 0.130 |
| Val78 | –93.15 | 0.026 | –89.80 | 0.149 | 3.34 | 0.152 |
| Gln79 | –93.38 | 0.017 | –84.78 | 0.090 | 8.60 | 0.092 |
| Tyr80 | –93.74 | 0.022 | –92.63 | 0.107 | 1.11 | 0.109 |
| Tyr81 | –94.26 | 0.179 | –90.41 | 0.605 | 3.84 | 0.631 |
| Met82 | –94.02 | 0.133 | –84.14 | 0.661 | 9.88 | 0.675 |
| Glu83 | –92.25 | 0.032 | –87.38 | 0.121 | 4.87 | 0.125 |
| His84 | –92.62 | 0.201 | –88.23 | 0.707 | 4.40 | 0.735 |
| Gln87 | –92.64 | 0.171 | –118.96 | 1.243 | –26.31 | 2.509 |
| Leu88 | –93.24 | 0.160 | –108.12 | 0.803 | –14.88 | 0.819 |
| Lys89 | –92.80 | 0.017 | –80.59 | 0.071 | 12.21 | 0.073 |
| Glu90 | –92.44 | 0.020 | –87.97 | 0.078 | 4.46 | 0.080 |
| Lys91 | –92.44 | 0.083 | –82.08 | 0.319 | 10.36 | 0.659 |
| Asn92 | –93.27 | 0.034 | –93.11 | 0.086 | 0.17 | 0.092 |
| Gly93 | –93.26 | 0.012 | –88.34 | 0.029 | 4.93 | 0.031 |
| Asp94 | –93.99 | 0.009 | –101.81 | 0.039 | –7.82 | 0.040 |
| Val95 | –92.81 | 0.012 | –85.09 | 0.107 | 7.72 | 0.107 |
| Ile96 | –93.20 | 0.023 | –70.66 | 0.078 | 22.54 | 0.082 |
| Glu97 | –92.66 | 0.016 | –69.26 | 0.223 | 23.40 | 0.223 |
| Leu98 | –93.20 | 0.027 | –71.80 | 0.084 | 21.40 | 0.088 |
| Lys99 | –92.40 | 0.025 | –84.88 | 0.106 | 7.52 | 0.109 |
| Tyr100 | –91.29 | 0.020 | –110.59 | 0.129 | –19.31 | 0.130 |
| Leu102 | –94.11 | 0.024 | –114.54 | 0.160 | –20.44 | 0.162 |
| Asn103 | –92.34 | 0.035 | –98.66 | 0.135 | –6.32 | 0.279 |

**
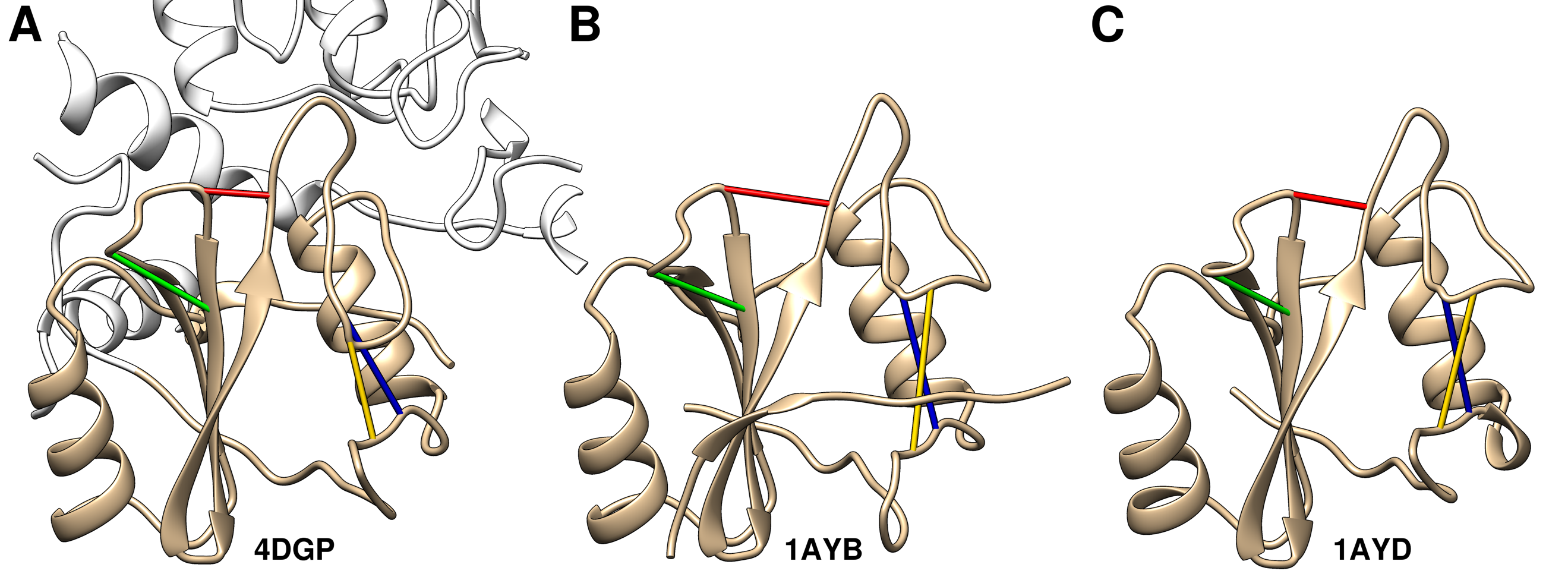
**

**Figure S1.** Cartoon representations of the N-SH2 domain in (A) the crystal structure of autoinhibited SHP2 (PDB ID 4DGP), (B) the crystal structure of isolated N-SH2 complexed with a high-affinity phosphopeptide (PDB ID 1AYB), (C) the crystal structure of isolated N-SH2 in apo form (*i.e.*, in the absence of a phosphopeptide, PDB ID 1AYD). Colored sticks represent the key interatomic distances used to quantify β-sheet spread (red), pY loop opening (green), binding cleft opening (yellow), and +5 site opening (blue). The PTP domain in autoinhibited SHP2 is depicted in white cartoon representation.

**
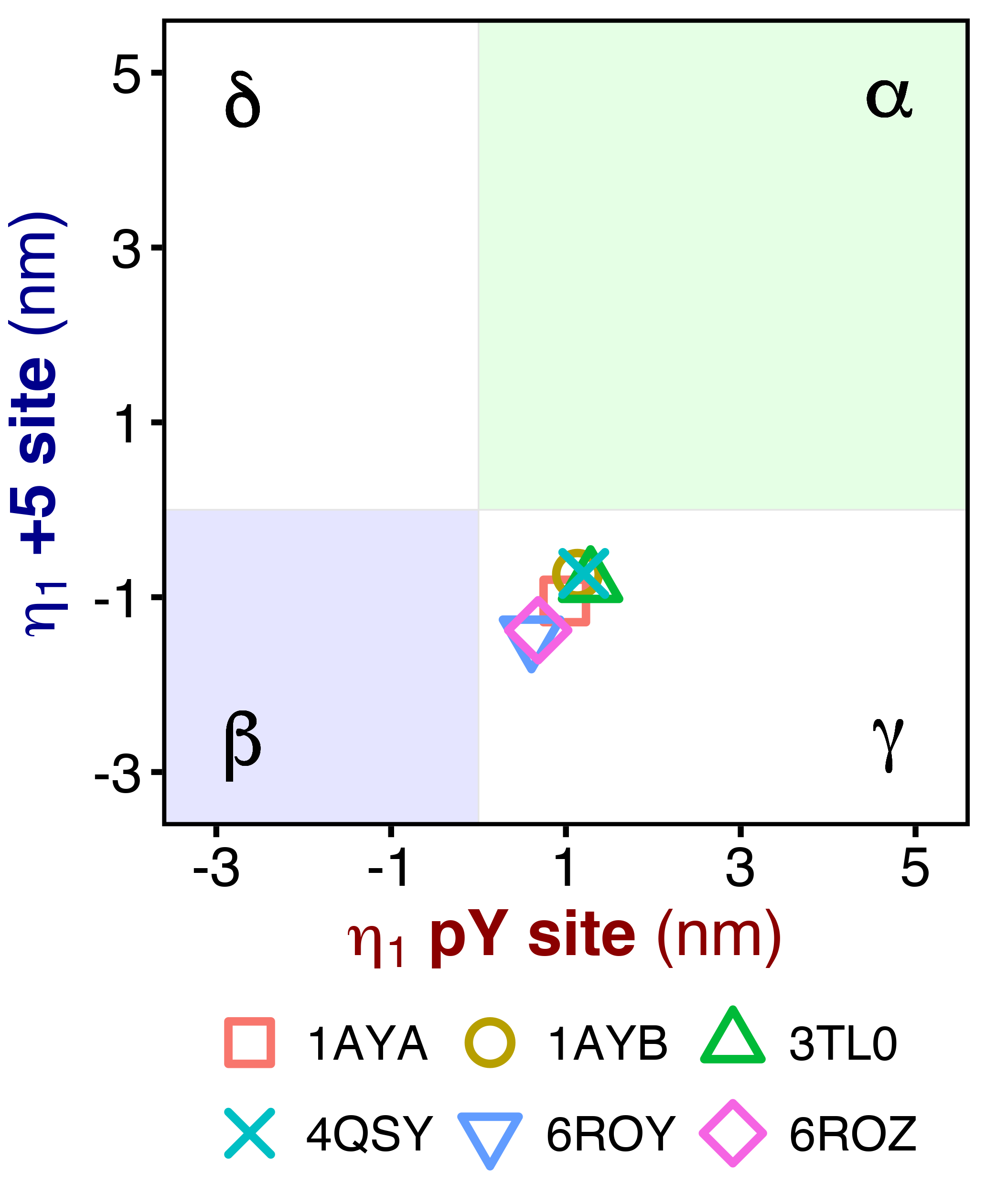
**

**Figure S2.** Projection of the crystal structures of the N-SH2 domain onto the PCA subvectors of the pY site (*x*-axis) and of the +5 site (*y*-axis). The structures belong to the isolated N-SH2 domain bound to human PDGFRB pY1009 peptide (1AYA), human IRS-1 pY895 peptide (1AYB), synthetic peptide RLNpYAQLWHR (3TL0), human GAB1 pY630 peptide (4QSY), PD-1 ITIM peptide (6ROY), and PD-1 ITSM peptide (6ROZ).

**
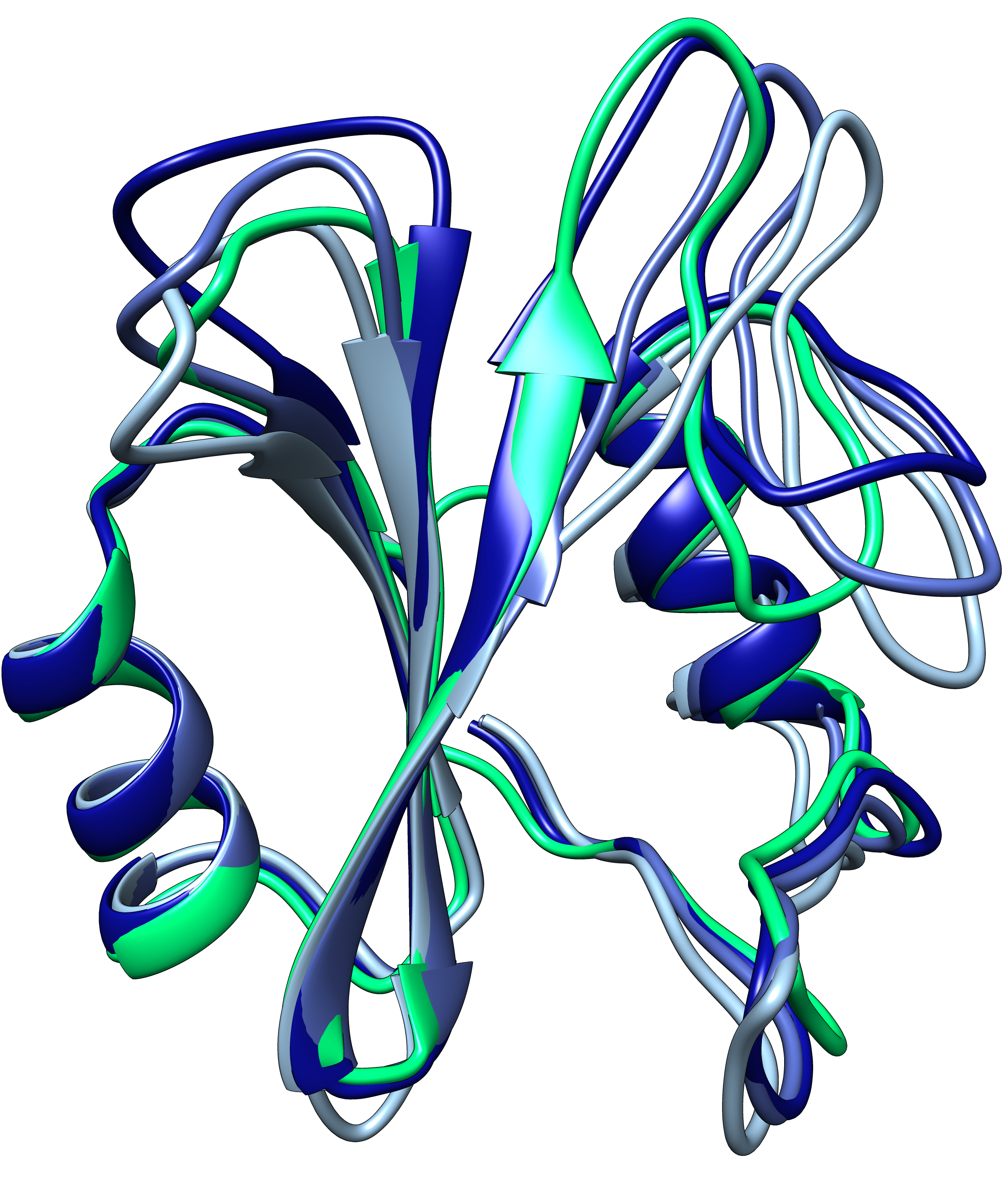
**

**Figure S3.** Overlay of the crystal structure of the N-SH2 domain in autoinhibited SHP2 (cartoon colored in green, PDB ID 4DGP) with structures representing the principal component of motion that describes the conformational transition from β to α (cartoon colored in shades of blue). The closure of the binding cleft in the 4DGP crystal structure is different from the closure of the +5 site, which is accomplished through two combined motions: *i)* the displacement of the EF loop towards the BG loop and *ii)* a parallel displacement of the EF loop relative to the BG loop with consequent torsion of the blocking loop. The closure of the binding cleft observed in 4DGP crystal structures only partially affects the +5 site principal component of motion detected by MD simulations in solution.

**
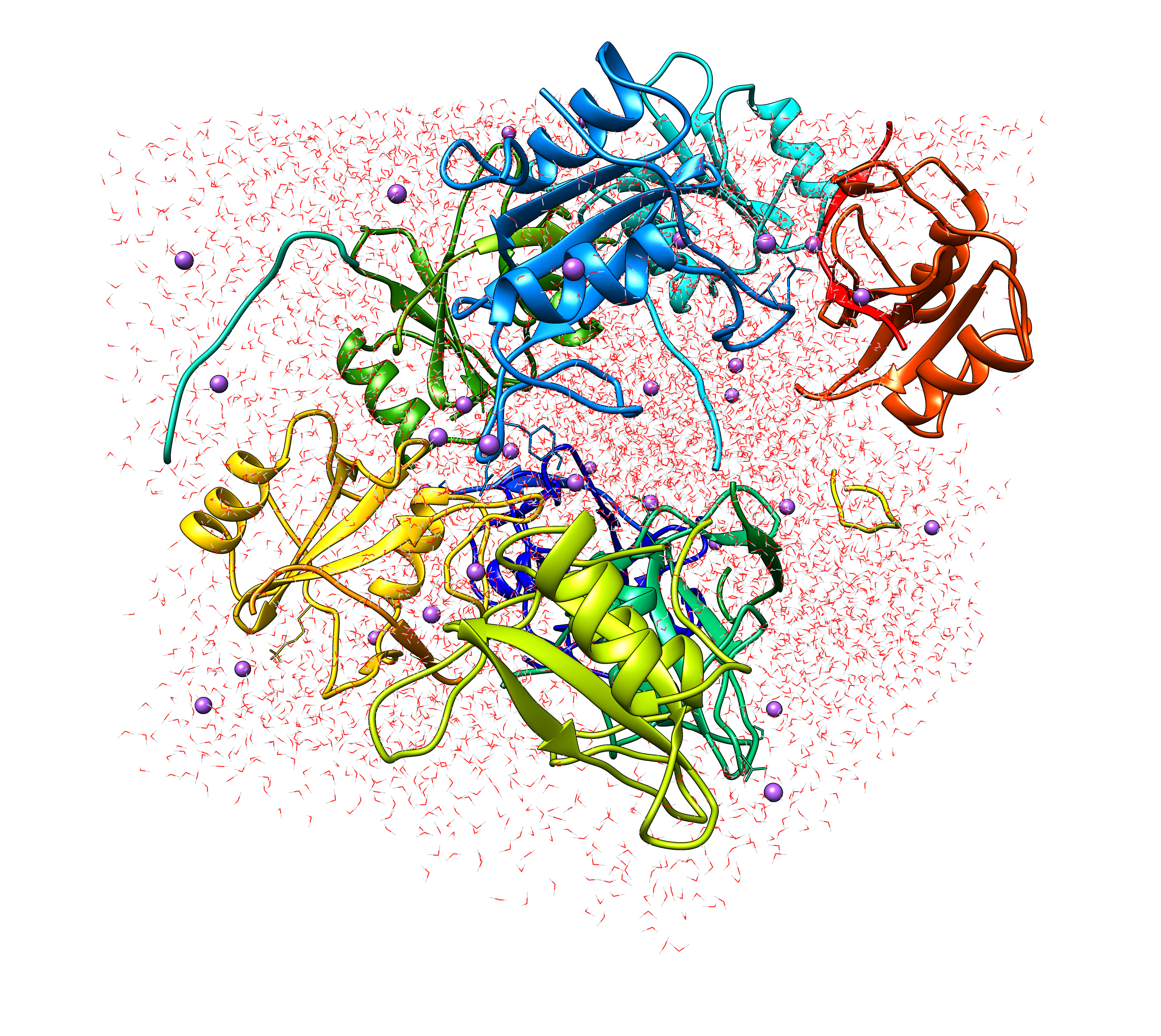
**

**Figure S4.** Simulation system of the N-SH2 domain complexed with the IRS-1 pY895 peptide in the crystallographic unit cell of the 1AYB structure. The system contains 8 N-SH2 copies shown in different colors. Water molecules are shown as red/white sticks and Na^+^ ions as purple spheres.

**
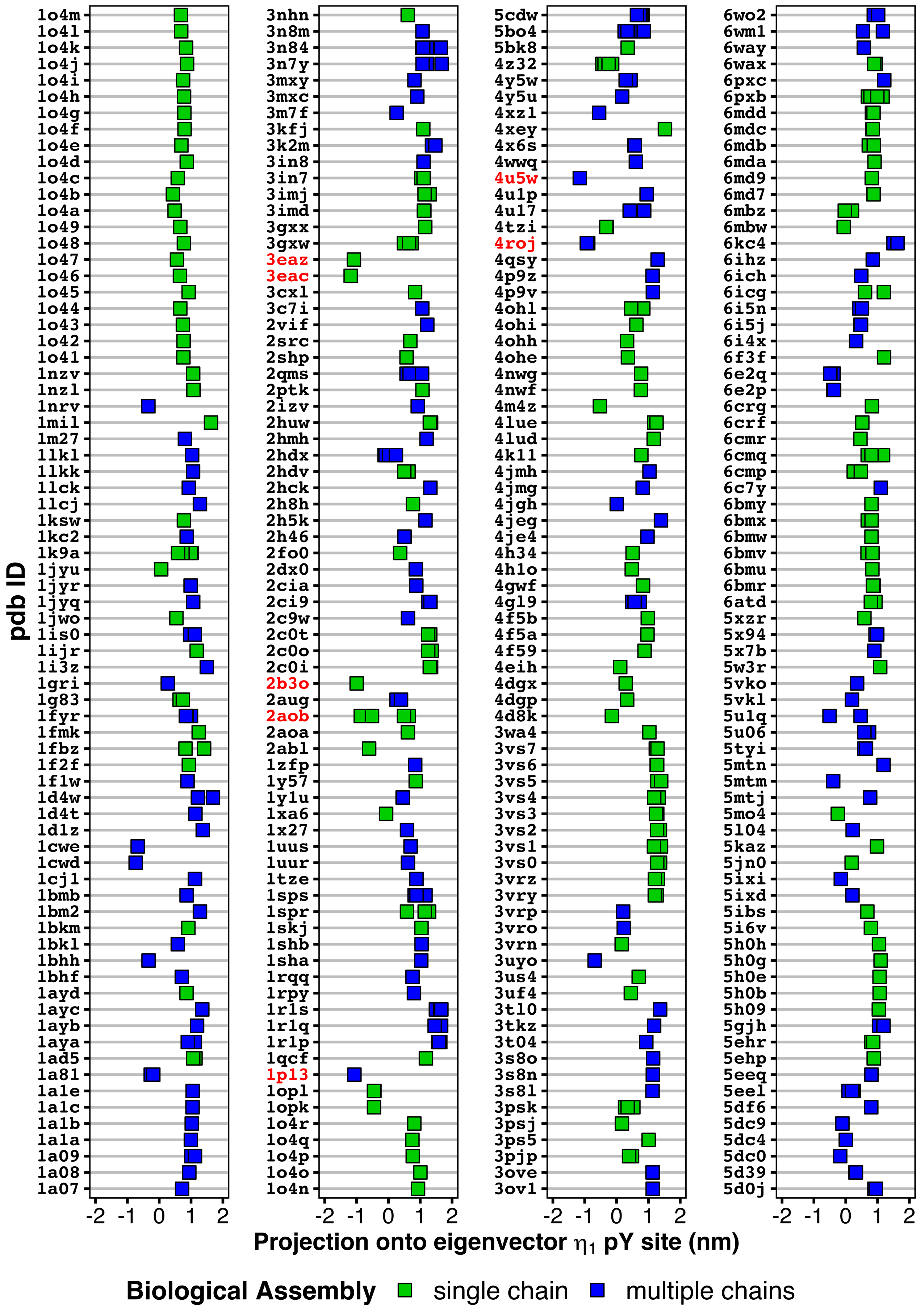
**

**Figure S5.** Projection of the SH2 domain structures onto the PCA subvector of the pY site for each crystal structure containing at least one SH2 domain (PROSITE entry PS50001) and sharing high sequence homology with *PTPN11* N-SH2. Calculations were performed on each biological assembly containing single (green) or multiple (blue) chains. Crystal structures in which the pY loop was completely open (η_1_ < –0.75) are highlighted in red.

**
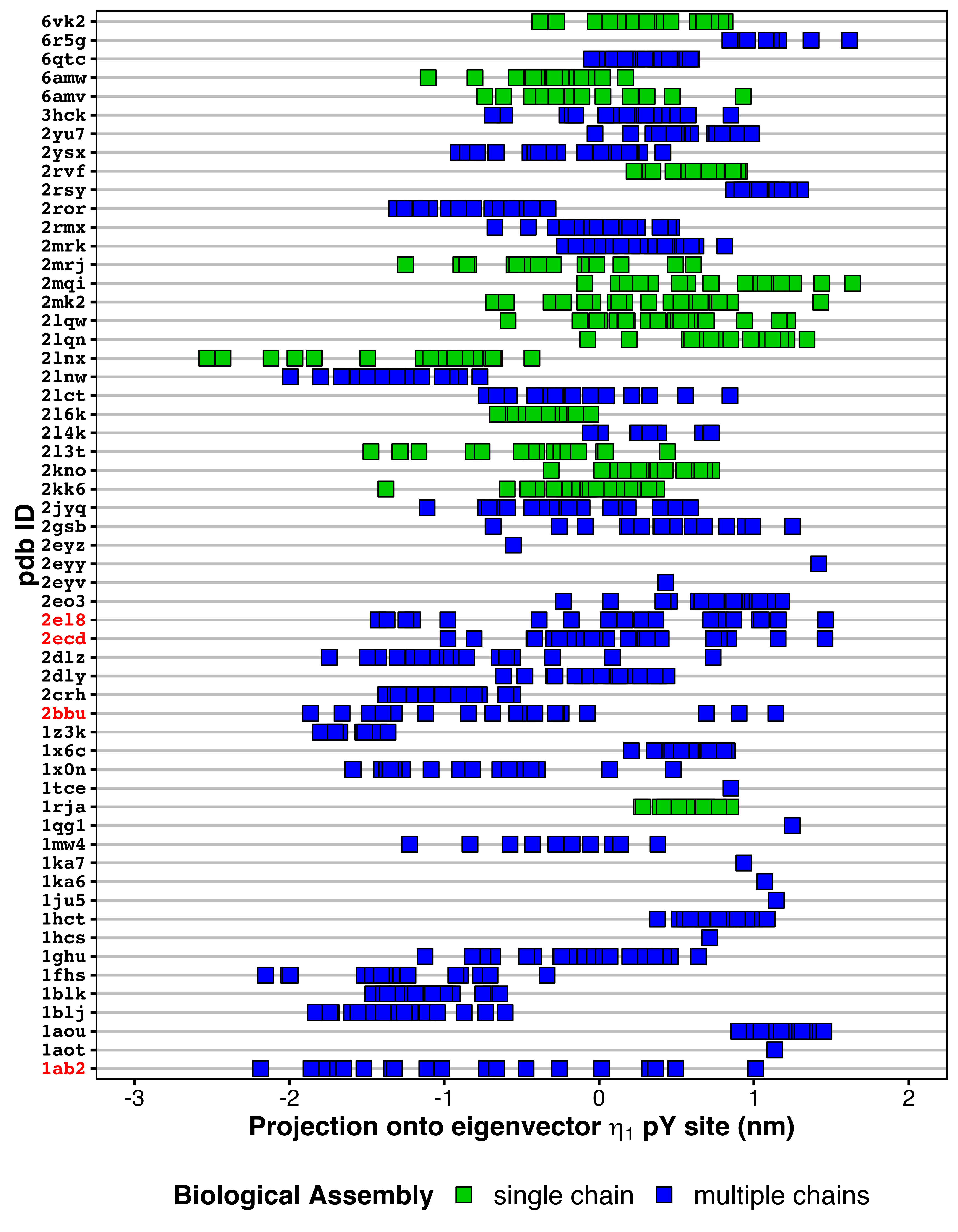
**

**Figure S6.** Projection of the SH2 domain structures onto the PCA subvector of the pY site for each NMR structure/model containing at least one SH2 domain (PROSITE entry PS50001) and sharing high sequence homology with *PTPN11* N-SH2. Calculations were performed on each model and biological assembly containing single (green) or multiple (blue) chains. NMR structures showing a complete opening/closure of the pY loop (η_1_ < –0.75 and η_1_ > 0.75) in one SH2 domain are highlighted in red.

**
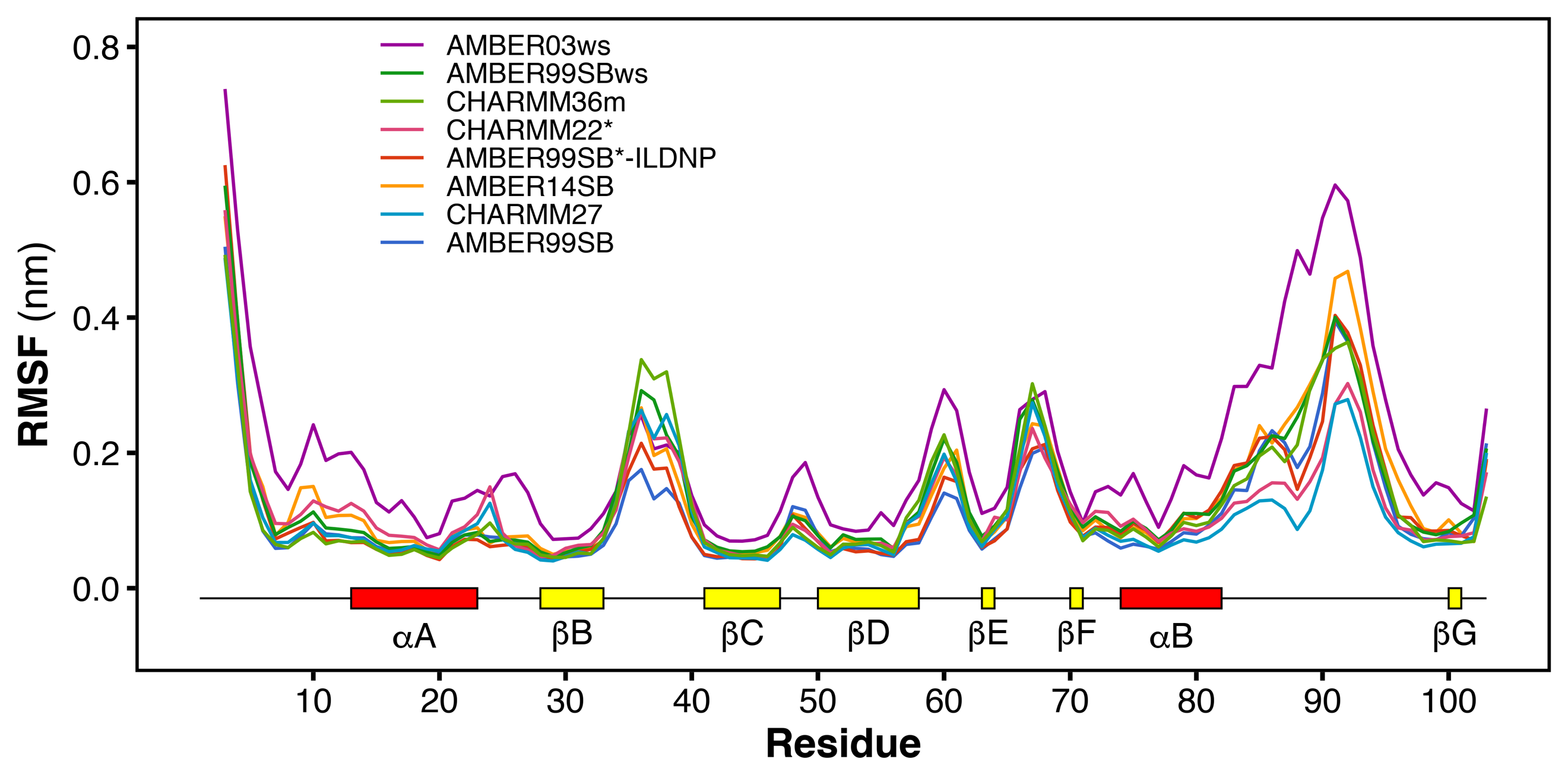
**

**Figure S7.** Root mean-square fluctuation (RMSF) of the Cα atoms in the N-SH2 domain determined for each force field. Secondary structures are indicated by red rectangles (α-helices), yellow rectangles (β-sheets), and solid lines (coil, other).

**
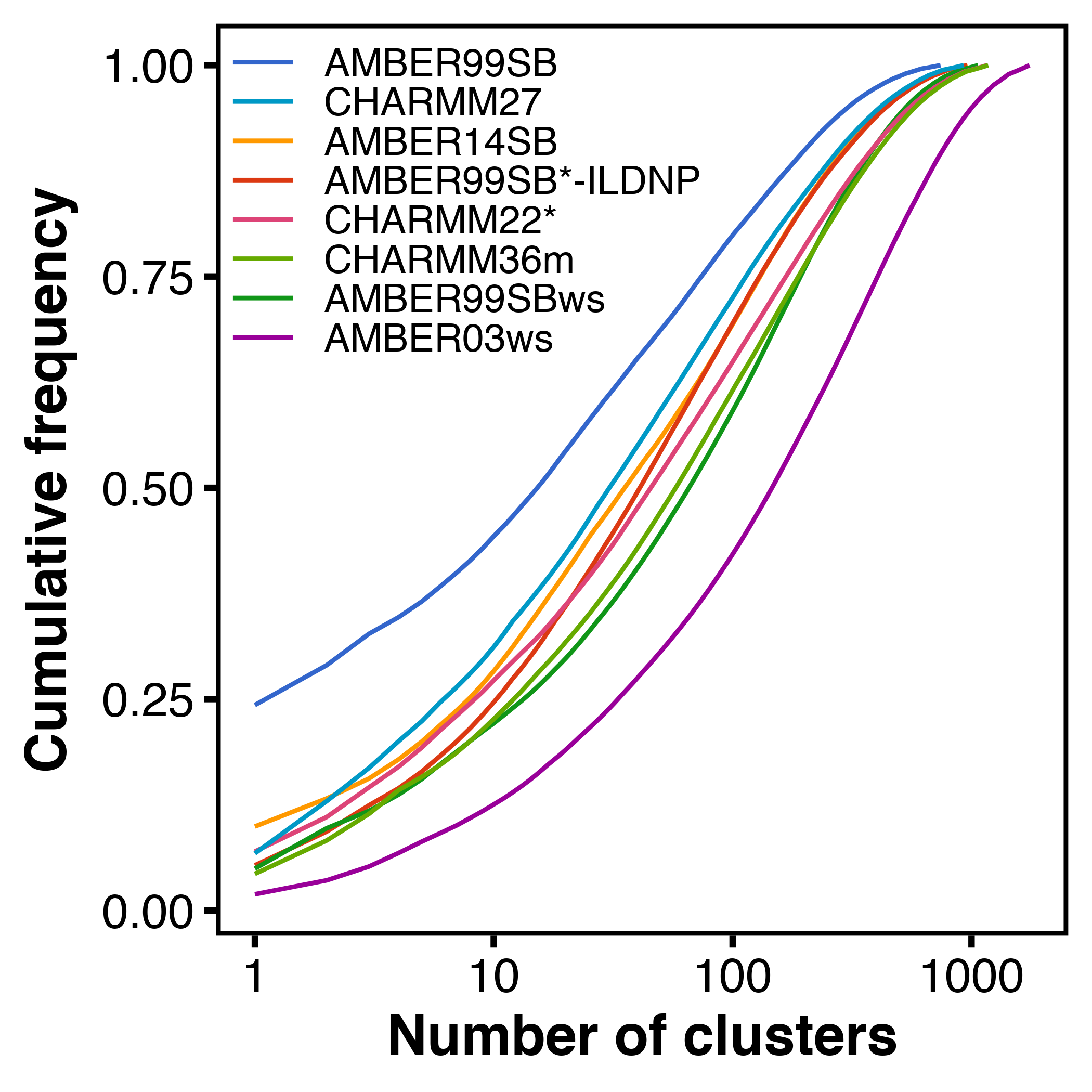
**

**Figure S8.** Empirical cumulative distribution of the N-SH2 conformations as a function of the number of clusters for each force field. Lower curves indicate more structurally diverse conformational ensembles, while higher curves indicate ensembles composed of fewer but better-defined states.

**
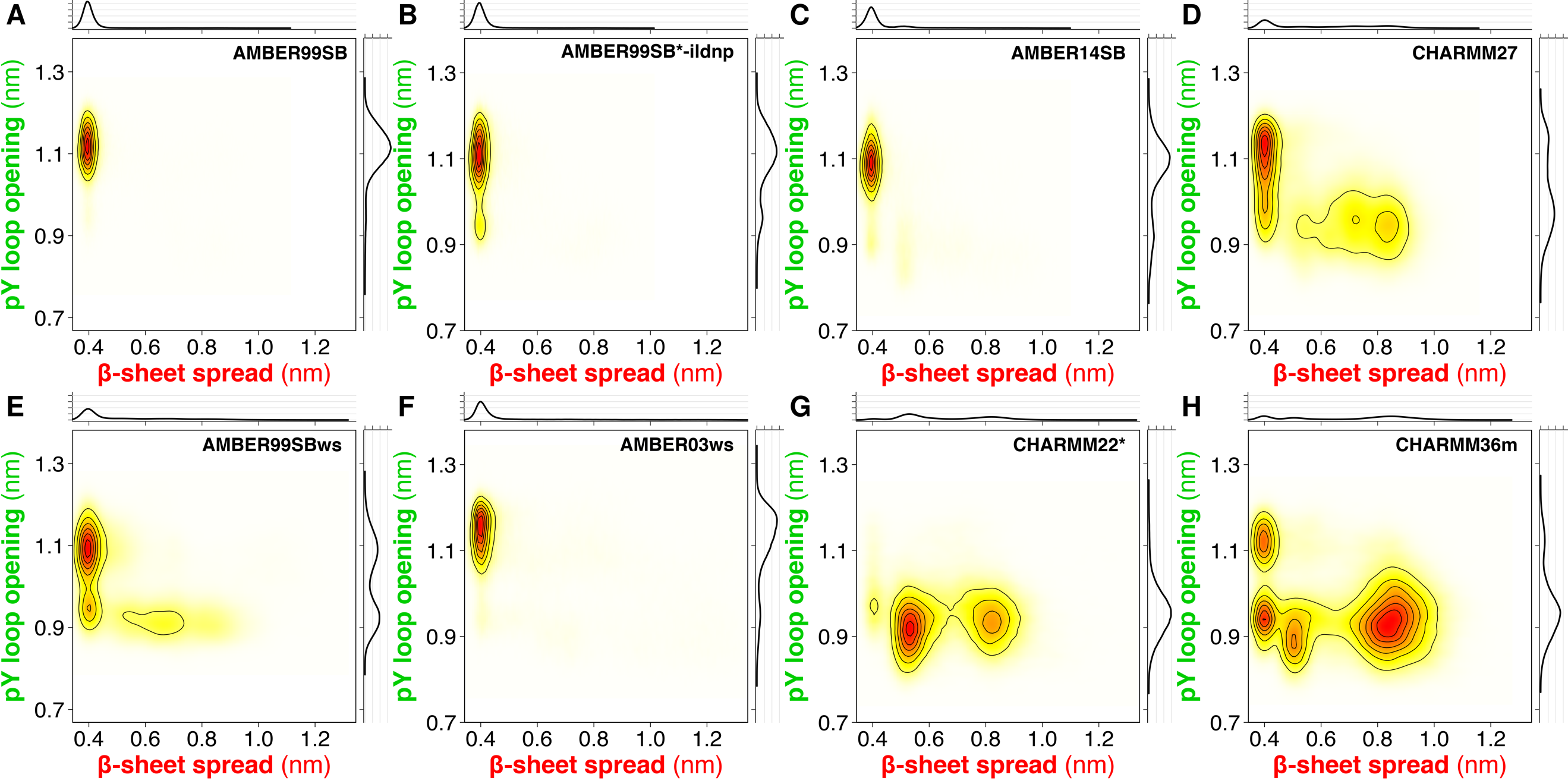
**

**Figure S9.** Bivariate probability distributions with respect to the β-sheet spread and the pY loop opening for the ensembles generated with each of eight different force fields, given in each panel. Univariate distributions with respect to the β-sheet spread and the pY loop opening are shown as axis projections along the respective axes.

**
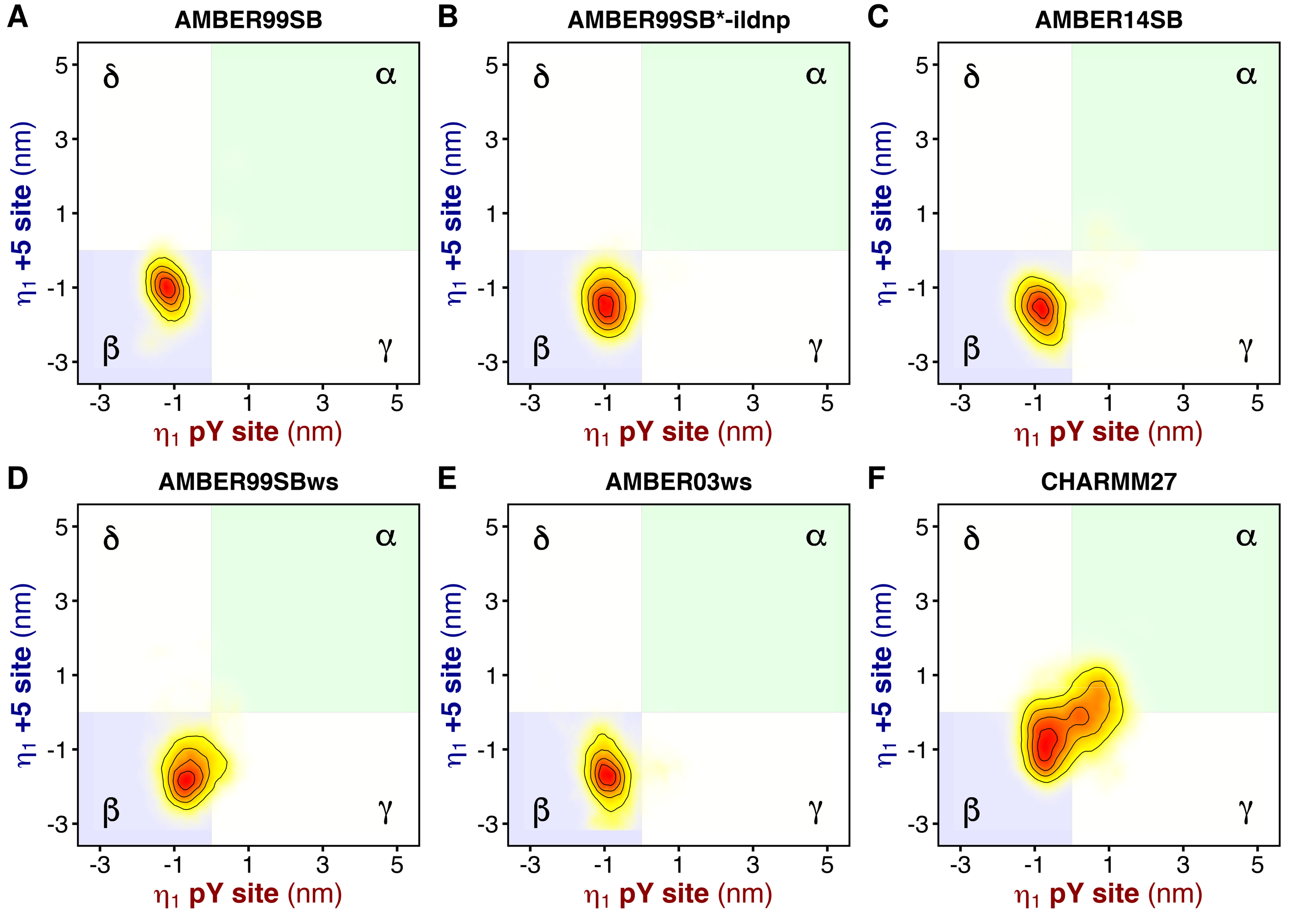
**

**Figure S10.** Projection of apo N-SH2 trajectories obtained from different force fields (subplot titles), projected onto the PCA subvectors of the pY site (*x*-axis) and of the +5 site (*y*-axis). The region corresponding to the α-state (pY loop closed, +5 site closed) is shaded in green, while the region corresponding to the β-state (pY loop open, +5 site open) is shaded in blue. The white regions indicate the γ-state (pY loop closed, +5 site open) and the δ-state (pY loop open, +5 site closed).

**
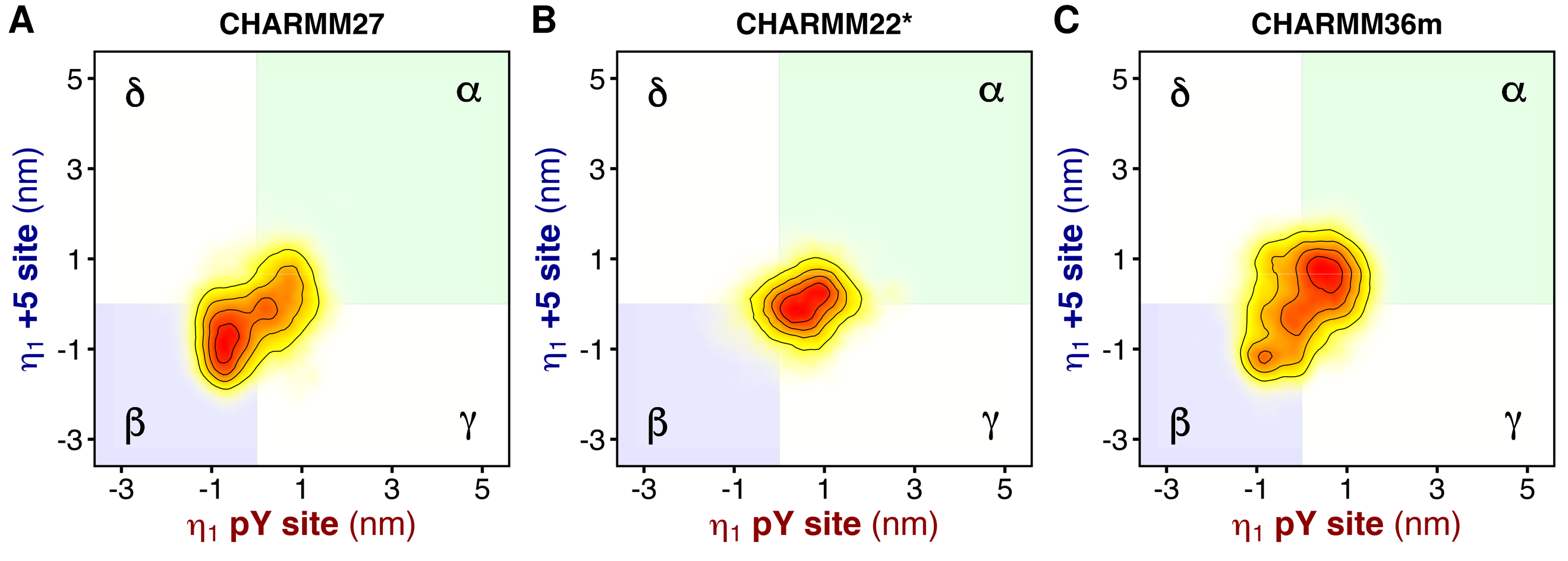
**

**Figure S11.** Projection of apo N-SH2 trajectories obtained from different CHARMM force fields (subplot titles), projected onto the PCA subvectors of the pY site (*x*-axis) and of the +5 site (*y*-axis). The region corresponding to the α-state (pY loop closed, +5 site closed) is shaded in green, while the region corresponding to the β-state (pY loop open, +5 site open) is shaded in blue. The white regions indicate the γ-state (pY loop closed, +5 site open) and the δ-state (pY loop open, +5 site closed).

**
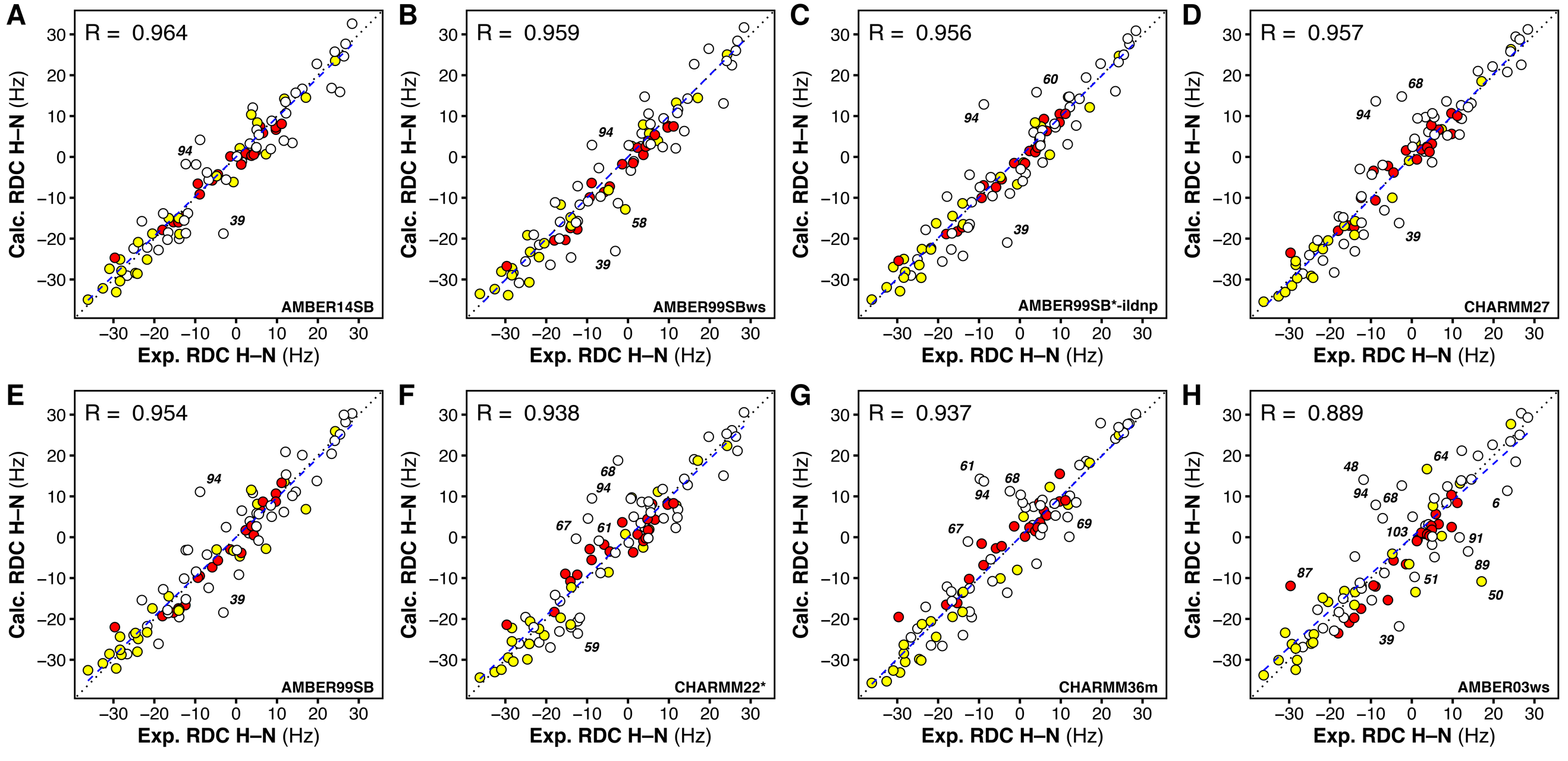
**

**Figure S12.** Correlation between the experimental H–N residual dipolar couplings (Exp. RDC H–N) and the back-calculated H–N residual dipolar couplings (Calc. RDC H–N) as obtained from the best fitting of 100 randomly selected conformations from the ensemble of the isolated unliganded N-SH2 in solution generated with each of the eight force fields. The RDC were fitted with a single SVD-derived alignment tensor. Pearson correlation coefficients *R* are shown in each panel. The points are colored according to the secondary structure of the first residue involved: red (α-helix), yellow (β-sheet), white (coil, other). The points with the highest deviation are marked with the sequence number of the first residue involved (for the location of the residue in the N-SH2 structure see Figure S13).

**
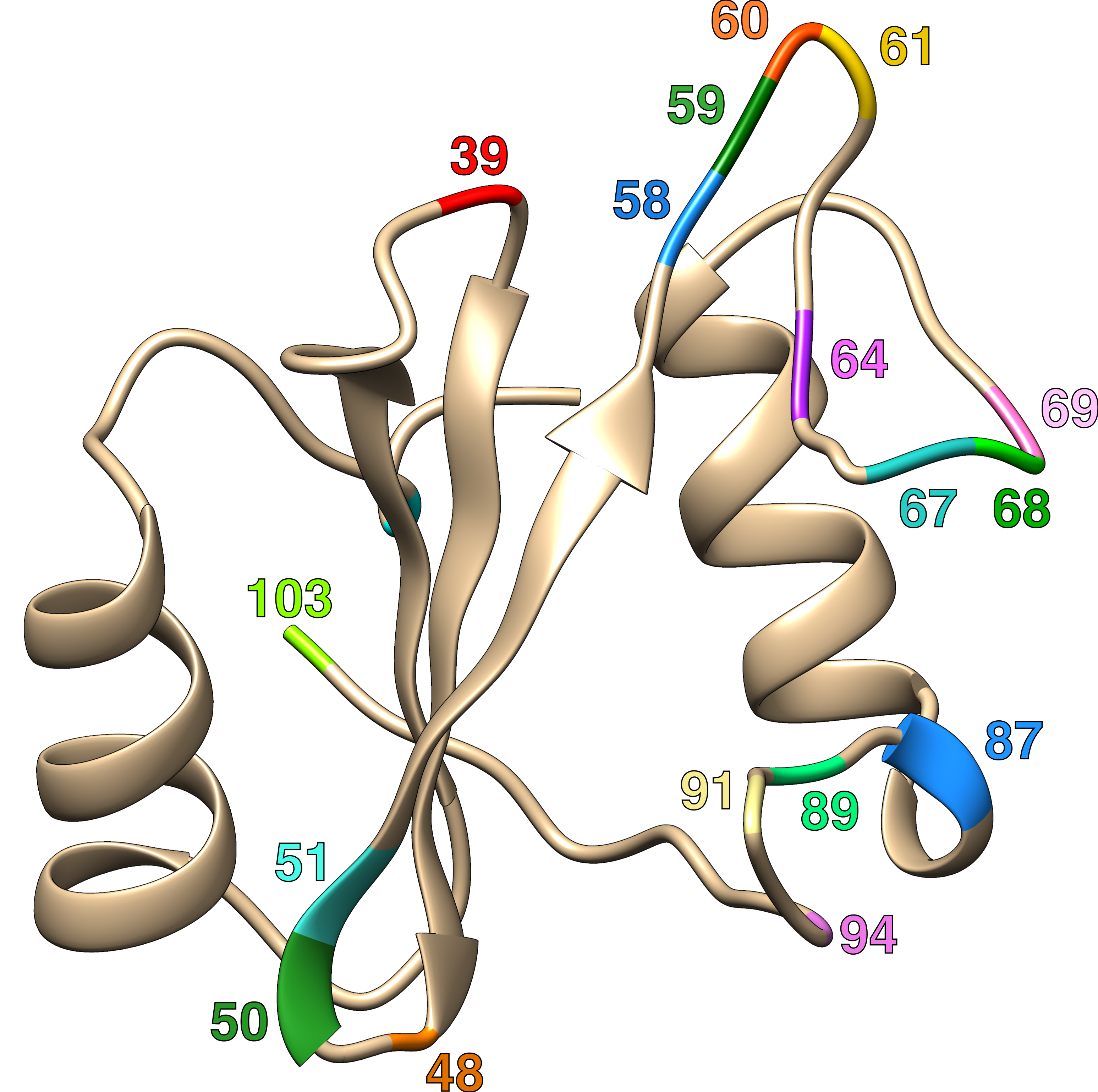
**

**Figure S13.** Cartoon representation of the crystal structure of the unbound N-SH2 domain (PDB ID 1AYD). The locations of the residues showing the highest deviation between experimental and calculated H–N RDCs, as reported in Figure S12, are highlighted in arbitrary colors.

**List of the PDB entries containing SH2 domains (PROSITE entry PS50001 in Dec 2021)**

1a07, 1a08, 1a09, 1a1a, 1a1b, 1a1c, 1a1e, 1a81, 1ab2, 1ad5, 1aot, 1aou, 1aya, 1ayb, 1ayc, 1ayd, 1bf5, 1bfi, 1bfj, 1bg1, 1bhf, 1bhh, 1bkl, 1bkm, 1blj, 1blk, 1bm2, 1bmb, 1cj1, 1csy, 1csz, 1cwd, 1cwe, 1d1z, 1d4t, 1d4w, 1f1w, 1f2f, 1fbz, 1fhs, 1fmk, 1fu5, 1fu6, 1fyr, 1g83, 1ghu, 1gri, 1h9o, 1hcs, 1hct, 1i3z, 1ijr, 1is0, 1ju5, 1jwo, 1jyq, 1jyr, 1jyu, 1k9a, 1ka6, 1ka7, 1kc2, 1ksw, 1lcj, 1lck, 1lkk, 1lkl, 1lui, 1luk, 1lum, 1lun, 1m27, 1m61, 1mil, 1mw4, 1nrv, 1nzl, 1nzv, 1o41, 1o42, 1o43, 1o44, 1o45, 1o46, 1o47, 1o48, 1o49, 1o4a, 1o4b, 1o4c, 1o4d, 1o4e, 1o4f, 1o4g, 1o4h, 1o4i, 1o4j, 1o4k, 1o4l, 1o4m, 1o4n, 1o4o, 1o4p, 1o4q, 1o4r, 1oo3, 1oo4, 1opk, 1opl, 1p13, 1pic, 1qad, 1qcf, 1qg1, 1r1p, 1r1q, 1r1s, 1rja, 1rpy, 1rqq, 1sha, 1shb, 1shd, 1skj, 1spr, 1sps, 1tce, 1tze, 1uur, 1uus, 1wqu, 1x0n, 1x27, 1x6c, 1xa6, 1y1u, 1y57, 1yvl, 1z3k, 1zfp, 2abl, 2aoa, 2aob, 2aug, 2b3o, 2bbu, 2c0i, 2c0o, 2c0t, 2c9w, 2ci8, 2ci9, 2cia, 2cr4, 2crh, 2cs0, 2dcr, 2dly, 2dlz, 2dm0, 2dvj, 2dx0, 2ecd, 2ekx, 2el8, 2eo3, 2eo6, 2eob, 2etz, 2eu0, 2eyv, 2eyy, 2eyz, 2fci, 2fo0, 2ge9, 2gsb, 2h46, 2h5k, 2h8h, 2hck, 2hdv, 2hdx, 2hmh, 2huw, 2iug, 2iuh, 2iui, 2izv, 2jyq, 2k79, 2k7a, 2kk6, 2kno, 2l3t, 2l4k, 2l6k, 2lct, 2lnw, 2lnx, 2lqn, 2lqw, 2mc1, 2mk2, 2mqi, 2mrj, 2mrk, 2oq1, 2ozo, 2pld, 2ple, 2pna, 2pnb, 2ptk, 2qms, 2rd0, 2rmx, 2ror, 2rsy, 2rvf, 2shp, 2src, 2vif, 2y3a, 2ysx, 2yu7, 3bkb, 3c7i, 3cbl, 3cd3, 3cwg, 3cxl, 3eac, 3eaz, 3gqi, 3gxw, 3gxx, 3hck, 3hhm, 3hiz, 3imd, 3imj, 3in7, 3in8, 3k2m, 3kfj, 3m7f, 3maz, 3mxc, 3mxy, 3n7y, 3n84, 3n8m, 3nhn, 3ov1, 3ove, 3pjp, 3pqz, 3ps5, 3psi, 3psj, 3psk, 3qwx, 3qwy, 3s8l, 3s8n, 3s8o, 3s9k, 3t04, 3tkz, 3tl0, 3uf4, 3us4, 3uyo, 3vrn, 3vro, 3vrp, 3vry, 3vrz, 3vs0, 3vs1, 3vs2, 3vs3, 3vs4, 3vs5, 3vs6, 3vs7, 3wa4, 4a55, 4d8k, 4dgp, 4dgx, 4e68, 4e93, 4eih, 4ey0, 4f59, 4f5a, 4f5b, 4fbn, 4fl2, 4fl3, 4gl9, 4gwf, 4h1o, 4h34, 4je4, 4jeg, 4jgh, 4jmg, 4jmh, 4jps, 4k11, 4k2r, 4k44, 4k45, 4l1b, 4l23, 4l2y, 4lud, 4lue, 4m4z, 4nwf, 4nwg, 4ohd, 4ohe, 4ohh, 4ohi, 4ohl, 4ovu, 4ovv, 4p9v, 4p9z, 4qsy, 4roj, 4tzi, 4u17, 4u1p, 4u5w, 4waf, 4wwq, 4x6s, 4xey, 4xi2, 4xz0, 4xz1, 4y5u, 4y5w, 4ykn, 4z32, 4zop, 5aul, 5bk8, 5bo4, 5cdw, 5d0j, 5d39, 5dc0, 5dc4, 5dc9, 5df6, 5eel, 5eeq, 5eg3, 5ehp, 5ehr, 5fi4, 5gjh, 5gji, 5h09, 5h0b, 5h0e, 5h0g, 5h0h, 5i6v, 5ibm, 5ibs, 5itd, 5ixd, 5ixi, 5jn0, 5kaz, 5l04, 5m6u, 5mo4, 5mtj, 5mtm, 5mtn, 5sw8, 5swg, 5swo, 5swp, 5swr, 5swt, 5sx8, 5sx9, 5sxa, 5sxb, 5sxc, 5sxd, 5sxe, 5sxf, 5sxi, 5sxj, 5sxk, 5tnw, 5to4, 5tq1, 5tqs, 5tyi, 5u06, 5u1q, 5uk8, 5ukj, 5ul1, 5vkl, 5vko, 5w3r, 5x7b, 5x94, 5xgh, 5xgi, 5xgj, 5xzr, 6amv, 6amw, 6atd, 6bmr, 6bmu, 6bmv, 6bmw, 6bmx, 6bmy, 6bn5, 6c7y, 6cmp, 6cmq, 6cmr, 6cms, 6crf, 6crg, 6e2p, 6e2q, 6f3f, 6gme, 6gmh, 6htf, 6i4x, 6i5j, 6i5n, 6icg, 6ich, 6ihz, 6jmf, 6kc4, 6mbw, 6mbz, 6md7, 6md9, 6mda, 6mdb, 6mdc, 6mdd, 6nct, 6njs, 6nuq, 6pbc, 6pxb, 6pxc, 6qhd, 6qtc, 6r5g, 6ted, 6tlc, 6ux2, 6vk2, 6wax, 6way, 6wcz, 6wm1, 6wo2

**List of the PDB entries (biological assemblies) containing SH2 domains**

1a07.1, 1a08.1, 1a08.2, 1a09.1, 1a09.2, 1a09.3, 1a1a.1, 1a1b.1, 1a1c.1, 1a1e.1, 1a81.1, 1a81.2, 1a81.3, 1a81.4, 1a81.5, 1a81.6, 1a81.7, 1a81.8, 1ab2.1, 1ad5.1, 1ad5.2, 1aot.1, 1aou.1, 1aya.1, 1aya.2, 1ayb.1, 1ayc.1, 1ayd.1, 1bf5.1, 1bfi.1, 1bfj.1, 1bg1.1, 1bhf.1, 1bhh.1, 1bkl.1, 1bkm.1, 1blj.1, 1blk.1, 1bm2.1, 1bm2.2, 1bmb.1, 1bmb.2, 1cj1.1, 1cj1.2, 1cj1.3, 1cj1.4, 1cj1.5, 1cj1.6, 1csy.1, 1csz.1, 1cwd.1, 1cwe.1, 1d1z.1, 1d1z.2, 1d4t.1, 1d4w.1, 1d4w.2, 1f1w.1, 1f1w.2, 1f1w.3, 1f2f.1, 1fbz.1, 1fbz.2, 1fhs.1, 1fmk.1, 1fu5.1, 1fu6.1, 1fyr.1, 1fyr.2, 1fyr.3, 1fyr.4, 1fyr.5, 1fyr.6, 1g83.1, 1g83.2, 1ghu.1, 1gri.1, 1h9o.1, 1hcs.1, 1hct.1, 1i3z.1, 1ijr.1, 1is0.1, 1is0.2, 1ju5.1, 1jwo.1, 1jyq.1, 1jyq.2, 1jyr.1, 1jyr.2, 1jyu.1, 1jyu.2, 1k9a.1, 1k9a.2, 1k9a.3, 1k9a.4, 1k9a.5, 1k9a.6, 1k9a.7, 1ka6.1, 1ka7.1, 1kc2.1, 1ksw.1, 1lcj.1, 1lck.1, 1lkk.1, 1lkl.1, 1lui.1, 1luk.1, 1lum.1, 1lun.1, 1m27.1, 1m61.1, 1mil.1, 1mw4.1, 1nrv.1, 1nzl.1, 1nzl.2, 1nzv.1, 1nzv.2, 1o41.1, 1o42.1, 1o43.1, 1o44.1, 1o45.1, 1o46.1, 1o47.1, 1o48.1, 1o49.1, 1o4a.1, 1o4b.1, 1o4c.1, 1o4d.1, 1o4e.1, 1o4f.1, 1o4g.1, 1o4h.1, 1o4i.1, 1o4j.1, 1o4k.1, 1o4l.1, 1o4m.1, 1o4n.1, 1o4o.1, 1o4p.1, 1o4q.1, 1o4r.1, 1oo3.1, 1oo4.1, 1opk.1, 1opl.1, 1opl.2, 1opl.3, 1p13.1, 1p13.2, 1pic.1, 1qad.1, 1qcf.1, 1qg1.1, 1r1p.1, 1r1p.2, 1r1p.3, 1r1p.4, 1r1q.1, 1r1q.2, 1r1q.3, 1r1s.1, 1r1s.2, 1r1s.3, 1r1s.4, 1r1s.5, 1r1s.6, 1rja.1, 1rpy.1, 1rpy.2, 1rqq.1, 1sha.1, 1shb.1, 1shd.1, 1skj.1, 1spr.1, 1spr.2, 1spr.3, 1spr.4, 1sps.1, 1sps.2, 1sps.3, 1tce.1, 1tze.1, 1tze.2, 1tze.3, 1tze.4, 1tze.5, 1uur.1, 1uus.1, 1wqu.1, 1x0n.1, 1x27.1, 1x6c.1, 1xa6.1, 1y1u.1, 1y1u.2, 1y57.1, 1yvl.1, 1yvl.2, 1yvl.3, 1yvl.4, 1z3k.1, 1zfp.1, 1zfp.2, 1zfp.3, 1zfp.4, 1zfp.5, 2abl.1, 2aoa.1, 2aoa.2, 2aoa.3, 2aob.1, 2aob.2, 2aob.3, 2aob.4, 2aob.5, 2aob.6, 2aug.1, 2aug.2, 2b3o.1, 2bbu.1, 2c0i.1, 2c0i.2, 2c0o.1, 2c0o.2, 2c0t.1, 2c0t.2, 2c9w.1, 2ci8.1, 2ci9.1, 2ci9.2, 2cia.1, 2cr4.1, 2crh.1, 2cs0.1, 2dcr.1, 2dly.1, 2dlz.1, 2dm0.1, 2dvj.1, 2dx0.1, 2dx0.2, 2dx0.3, 2ecd.1, 2ekx.1, 2el8.1, 2eo3.1, 2eo6.1, 2eob.1, 2etz.1, 2eu0.1, 2eyv.1, 2eyy.1, 2eyz.1, 2fci.1, 2fo0.1, 2ge9.1, 2gsb.1, 2h46.1, 2h46.2, 2h5k.1, 2h8h.1, 2hck.1, 2hdv.1, 2hdv.2, 2hdx.1, 2hdx.2, 2hdx.3, 2hdx.4, 2hdx.5, 2hdx.6, 2hmh.1, 2huw.1, 2huw.2, 2iug.1, 2iuh.1, 2iui.1, 2iui.2, 2izv.1, 2jyq.1, 2k79.1, 2k7a.1, 2kk6.1, 2kno.1, 2l3t.1, 2l4k.1, 2l6k.1, 2lct.1, 2lnw.1, 2lnx.1, 2lqn.1, 2lqw.1, 2mc1.1, 2mk2.1, 2mqi.1, 2mrj.1, 2mrk.1, 2oq1.1, 2ozo.1, 2pld.1, 2ple.1, 2pna.1, 2pnb.1, 2ptk.1, 2qms.1, 2qms.2, 2qms.3, 2qms.4, 2rd0.1, 2rmx.1, 2ror.1, 2rsy.1, 2rvf.1, 2shp.1, 2shp.2, 2src.1, 2vif.1, 2y3a.1, 2ysx.1, 2yu7.1, 3bkb.1, 3c7i.1, 3cbl.1, 3cd3.1, 3cwg.1, 3cwg.2, 3cxl.1, 3eac.1, 3eaz.1, 3gqi.1, 3gxw.1, 3gxw.2, 3gxw.3, 3gxw.4, 3gxx.1, 3gxx.2, 3gxx.3, 3gxx.4, 3hck.1, 3hhm.1, 3hiz.1, 3imd.1, 3imd.2, 3imd.3, 3imj.1, 3imj.2, 3imj.3, 3in7.1, 3in7.2, 3in7.3, 3in8.1, 3k2m.1, 3k2m.2, 3kfj.1, 3kfj.2, 3m7f.1, 3maz.1, 3mxc.1, 3mxy.1, 3n7y.1, 3n7y.2, 3n7y.3, 3n7y.4, 3n7y.5, 3n84.1, 3n84.2, 3n84.3, 3n84.4, 3n84.5, 3n84.6, 3n84.7, 3n84.8, 3n84.9, 3n8m.1, 3n8m.2, 3nhn.1, 3ov1.1, 3ov1.2, 3ove.1, 3ove.2, 3pjp.1, 3pjp.2, 3pjp.3, 3pqz.1, 3pqz.2, 3ps5.1, 3ps5.2, 3psi.1, 3psj.1, 3psk.1, 3psk.2, 3psk.3, 3psk.4, 3psk.5, 3psk.6, 3qwx.1, 3qwy.1, 3qwy.2, 3s8l.1, 3s8n.1, 3s8n.2, 3s8o.1, 3s9k.1, 3s9k.2, 3t04.1, 3t04.2, 3t04.3, 3tkz.1, 3tl0.1, 3uf4.1, 3us4.1, 3us4.2, 3uyo.1, 3vrn.1, 3vro.1, 3vrp.1, 3vry.1, 3vry.2, 3vrz.1, 3vrz.2, 3vs0.1, 3vs0.2, 3vs1.1, 3vs1.2, 3vs2.1, 3vs2.2, 3vs3.1, 3vs3.2, 3vs4.1, 3vs4.2, 3vs5.1, 3vs5.2, 3vs6.1, 3vs6.2, 3vs7.1, 3vs7.2, 3wa4.1, 3wa4.2, 3wa4.3, 4a55.1, 4d8k.1, 4dgp.1, 4dgx.1, 4e68.1, 4e93.1, 4eih.1, 4ey0.1, 4ey0.2, 4ey0.3, 4ey0.4, 4f59.1, 4f5a.1, 4f5b.1, 4fbn.1, 4fl2.1, 4fl3.1, 4gl9.1, 4gl9.2, 4gl9.3, 4gl9.4, 4gwf.1, 4gwf.2, 4h1o.1, 4h34.1, 4je4.1, 4jeg.1, 4jgh.1, 4jgh.2, 4jmg.1, 4jmh.1, 4jps.1, 4k11.1, 4k2r.1, 4k44.1, 4k44.2, 4k45.1, 4l1b.1, 4l23.1, 4l2y.1, 4lud.1, 4lud.2, 4lue.1, 4lue.2, 4m4z.1, 4nwf.1, 4nwf.2, 4nwg.1, 4nwg.2, 4ohd.1, 4ohe.1, 4ohh.1, 4ohi.1, 4ohl.1, 4ohl.2, 4ovu.1, 4ovv.1, 4p9v.1, 4p9z.1, 4p9z.2, 4qsy.1, 4roj.1, 4roj.2, 4roj.3, 4tzi.1, 4tzi.2, 4u17.1, 4u17.2, 4u1p.1, 4u5w.1, 4waf.1, 4wwq.1, 4x6s.1, 4x6s.2, 4xey.1, 4xey.2, 4xi2.1, 4xz0.1, 4xz1.1, 4y5u.1, 4y5w.1, 4y5w.2, 4ykn.1, 4z32.1, 4z32.2, 4z32.3, 4z32.4, 4z32.5, 4z32.6, 4z32.7, 4z32.8, 4zop.1, 5aul.1, 5bk8.1, 5bo4.1, 5bo4.2, 5bo4.3, 5bo4.4, 5bo4.5, 5bo4.6, 5cdw.1, 5cdw.2, 5cdw.3, 5cdw.4, 5cdw.5, 5cdw.6, 5cdw.7, 5cdw.8, 5d0j.1, 5d0j.2, 5d39.1, 5d39.2, 5d39.3, 5dc0.1, 5dc4.1, 5dc9.1, 5df6.1, 5eel.1, 5eel.2, 5eel.3, 5eel.4, 5eel.5, 5eel.6, 5eeq.1, 5eeq.2, 5eg3.1, 5ehp.1, 5ehp.2, 5ehr.1, 5ehr.2, 5fi4.1, 5gjh.1, 5gjh.2, 5gji.1, 5h09.1, 5h0b.1, 5h0e.1, 5h0g.1, 5h0h.1, 5i6v.1, 5i6v.2, 5ibm.1, 5ibm.2, 5ibs.1, 5ibs.2, 5itd.1, 5ixd.1, 5ixi.1, 5jn0.1, 5kaz.1, 5l04.1, 5m6u.1, 5mo4.1, 5mtj.1, 5mtm.1, 5mtn.1, 5sw8.1, 5swg.1, 5swo.1, 5swp.1, 5swr.1, 5swt.1, 5sx8.1, 5sx9.1, 5sxa.1, 5sxb.1, 5sxc.1, 5sxd.1, 5sxe.1, 5sxf.1, 5sxi.1, 5sxj.1, 5sxk.1, 5tnw.1, 5tnw.2, 5to4.1, 5tq1.1, 5tqs.1, 5tqs.2, 5tqs.3, 5tqs.4, 5tyi.1, 5tyi.2, 5u06.1, 5u06.2, 5u1q.1, 5u1q.2, 5uk8.1, 5ukj.1, 5ul1.1, 5vkl.1, 5vko.1, 5w3r.1, 5x7b.1, 5x94.1, 5x94.2, 5xgh.1, 5xgi.1, 5xgj.1, 5xzr.1, 6amv.1, 6amw.1, 6atd.1, 6atd.2, 6bmr.1, 6bmr.2, 6bmu.1, 6bmu.2, 6bmv.1, 6bmv.2, 6bmw.1, 6bmw.2, 6bmx.1, 6bmx.2, 6bmy.1, 6bmy.2, 6bn5.1, 6bn5.2, 6c7y.1, 6cmp.1, 6cmp.2, 6cmq.1, 6cmq.2, 6cmq.3, 6cmq.4, 6cmr.1, 6cms.1, 6crf.1, 6crf.2, 6crg.1, 6crg.2, 6e2p.1, 6e2p.2, 6e2q.1, 6e2q.2, 6e2q.3, 6e2q.4, 6f3f.1, 6gme.1, 6gme.2, 6gmh.1, 6htf.1, 6i4x.1, 6i5j.1, 6i5j.2, 6i5n.1, 6i5n.2, 6icg.1, 6icg.2, 6ich.1, 6ihz.1, 6jmf.1, 6kc4.1, 6kc4.2, 6kc4.3, 6kc4.4, 6kc4.5, 6kc4.6, 6mbw.1, 6mbw.2, 6mbz.1, 6mbz.2, 6md7.1, 6md7.2, 6md9.1, 6md9.2, 6mda.1, 6mda.2, 6mdb.1, 6mdb.2, 6mdc.1, 6mdc.2, 6mdd.1, 6mdd.2, 6nct.1, 6njs.1, 6nuq.1, 6pbc.1, 6pxb.1, 6pxb.2, 6pxb.3, 6pxb.4, 6pxb.5, 6pxb.6, 6pxc.1, 6qhd.1, 6qtc.1, 6r5g.1, 6ted.1, 6tlc.1, 6ux2.1, 6vk2.1, 6wax.1, 6wax.2, 6way.1, 6wcz.1, 6wm1.1, 6wm1.2, 6wo2.1, 6wo2.2

**Parameterization history of the force fields used in this work**

**AMBER99SB.** This force field was developed as an improvement of AMBER99 (ff99) [1]. The φ/ψ dihedral terms were derived by fitting the energies of multiple conformations of glycine and alanine tetrapeptides from high-level *ab initio* quantum mechanical calculations [2]. Compared to ff99, this new parameter set achieved a better balance of secondary structure elements as judged by the improved distribution of backbone dihedrals for glycine and alanine with respect to PDB survey data [2]. The parameter set also accomplished improved agreement with published experimental data for conformational preferences of short alanine peptides and better accord with experimental NMR relaxation data of test protein systems [2].

**AMBER99SB*-ildnp.** This force field is the combination of the ff99SB* force field [3] with the ILDN modifications [4] and the improved proline and 4-hydroxyproline parameters [5]. Torsional backbone energy corrections were introduced in ff99SB to reproduce the fraction of α-helix measured in short peptides at 300 K [3]; the validation of the backbone-corrected ff99SB* force field produced results in excellent agreement with nuclear magnetic resonance experiments for folded proteins and short peptides not used in the optimization [3]. However, the rotamer distribution of Ile, Leu, Asp, Asn in standard ff99SB differed the most from expectations based on Protein Data Bank statistics [4]. For this reason, sidechain torsion potentials of those four amino acid types were optimized in order to match new, high-level quantum-mechanical calculations [4]. These modifications exhibited considerably better agreement with the NMR data [4]. The motional frequency of the Pro ring interconversion predicted by standard ff99SB was about six times faster than the experimental value [5]; improved Pro and Hyp parameters were derived from fittings of experimental correlation times and NMR J-couplings [5].

**AMBER14SB.** This force field was developed by means of a complete refit of all amino acids with the aim of improving the accuracy of the backbone and side chain parameters relative to ff99SB [6].

**AMBER99SBws.** This force field was developed starting from the ff99SB*-ILDN-q force field, which was a combination of ff99SB* force field [3] with the ILDN modifications [4], and the backbone charge modifications for the charged residues Arg, Asp, Glu, and Lys [7]. The backbone charge modifications were introduced in order to obtain identical backbone charges for all residues and to better reproduce the experimental α-helix propensity [7]; where necessary, side-chain torsion angles for the charged residues were refitted [7]. To allow usage in combination with the TIP4P/2005 water model [8], small adjustments were made to the torsional backbone energy corrections (*) [9]. Protein-water interactions were scaled (s) [9].

**AMBER03ws.** This force field was developed starting from the ff03* force field, which was itself a modification of ff03 to better reproduce α-helical propensity [3]. In order to be used in combination with the TIP4P/2005 water model [8], small adjustments were introduced in the torsional backbone energy corrections (*) [9]. Protein-water interactions were scaled (s) [9].

**CHARMM27.** Version of the CHARMM force field released in 1999 [10]. For purely protein systems, the force field is equivalent to CHARMM22 with a grid-based correction, called CMAP, for the φ/ψ dihedral potential [11].

**CHARMM22*.** This force field was developed starting from CHARMM22+CMAP (termed CHARMM27) [11]. In order to obtain a force field with an improved helix-coil balance, new backbone torsion terms replaced the CMAP correction for all residues (apart from Gly and Pro) [12]. The partial charges for Asp, Glu and Arg side chains, as well as the χ_1_ and χ_2_ torsion terms for the Asp side chain, were modified to provide a more accurate description of salt-bridge interactions [12].

**CHARMM36m.** This force field is a refinement of CHARMM36 [13] with improved accuracy in generating polypeptide backbone conformational ensembles for intrinsically disordered peptides and proteins [14]. The CHARMM36 force field was developed to improve helix-coil balance [13]. The common (non-Gly, non-Pro) backbone CMAP potentials were refined against experimental solution NMR data for weakly structured peptides, resulting in a rebalancing of the energies of the α-helix and extended regions of the Ramachandran map, correcting the α-helical bias of CHARMM22+CMAP [13]. The Gly and Pro CMAPs were refitted to more accurate quantum-mechanical energy surfaces [13]. Sidechain torsion parameters were optimized by fitting to backbone-dependent quantum-mechanical energy surfaces, followed by additional empirical optimization based on NMR scalar couplings for unfolded proteins [13].

**REFERENCES**

1. Wang J, Cieplak P, Kollman PA (2000) How well does a restrained electrostatic potential (RESP) model perform in calculating conformational energies of organic and biological molecules? J Comput Chem 21: 1049-74.

2. Hornak V, Abel R, Okur A, Strockbine B, Roitberg A, Simmerling C (2006) Comparison of multiple Amber force fields and development of improved protein backbone parameters. Proteins 65: 712-25.

3. Best RB, Hummer G (2009) Optimized Molecular Dynamics Force Fields Applied to the Helix−Coil Transition of Polypeptides. J Phys Chem B 113: 9004-15.

4. Lindorff-Larsen K, Piana S, Palmo K, Maragakis P, Klepeis JL, Dror RO, et al. (2010) Improved side-chain torsion potentials for the Amber ff99SB protein force field. Proteins 78: 1950-8.

5. Aliev AE, Kulke M, Khaneja HS, Chudasama V, Sheppard TD, Lanigan RM (2014) Motional timescale predictions by molecular dynamics simulations: Case study using proline and hydroxyproline sidechain dynamics. Proteins 82: 195-215.

6. Maier JA, Martinez C, Kasavajhala K, Wickstrom L, Hauser KE, Simmerling C (2015) ff14SB: Improving the Accuracy of Protein Side Chain and Backbone Parameters from ff99SB. J Chem Theory Comput 11: 3696-713.

7. Best Robert B, de Sancho D, Mittal J (2012) Residue-Specific α-Helix Propensities from Molecular Simulation. Biophys J 102: 1462-7.

8. Abascal JLF, Vega C (2005) A general purpose model for the condensed phases of water: TIP4P/2005. J Chem Phys 123: 234505.

9. Best RB, Zheng W, Mittal J (2014) Balanced Protein–Water Interactions Improve Properties of Disordered Proteins and Non-Specific Protein Association. J Chem Theory Comput 10: 5113-24.

10. MacKerell AD, Bashford D, Bellott M, Dunbrack RL, Evanseck JD, Field MJ, et al. (1998) All-Atom Empirical Potential for Molecular Modeling and Dynamics Studies of Proteins. J Phys Chem B 102: 3586-616.

11. Mackerell Jr AD, Feig M, Brooks III CL (2004) Extending the treatment of backbone energetics in protein force fields: Limitations of gas-phase quantum mechanics in reproducing protein conformational distributions in molecular dynamics simulations. J Comput Chem 25: 1400-15.

12. Piana S, Lindorff-Larsen K, Shaw David E (2011) How Robust Are Protein Folding Simulations with Respect to Force Field Parameterization? Biophys J 100: L47-L9.

13. Best RB, Zhu X, Shim J, Lopes PEM, Mittal J, Feig M, et al. (2012) Optimization of the Additive CHARMM All-Atom Protein Force Field Targeting Improved Sampling of the Backbone ϕ, ψ and Side-Chain χ1 and χ2 Dihedral Angles. J Chem Theory Comput 8: 3257-73.

14. Huang J, Rauscher S, Nawrocki G, Ran T, Feig M, de Groot BL, et al. (2017) CHARMM36m: an improved force field for folded and intrinsically disordered proteins. Nat Methods 14: 71-3.
